# Speciation Theory of Carcinogenesis Explains Karyotypic Individuality and Long Latencies of Cancers

**DOI:** 10.3390/genes9080402

**Published:** 2018-08-09

**Authors:** Ankit Hirpara, Mathew Bloomfield, Peter Duesberg

**Affiliations:** 1 Department of Molecular and Cell Biology, Donner Laboratory, University of California at Berkeley, Berkeley, CA 94720, USA; ankit_hirpara1@berkeley.edu; 2 Department of Natural Sciences and Mathematics, Dominican University of California, San Rafael, CA 94 901, USA; mbloomfield@berkeley.edu

**Keywords:** heritability, quasi-clonality, heterogeneity in clonal margins, single-step carcinogenesis, low probability of karyotypic speciation

## Abstract

It has been known for over 100 years that cancers have individual karyotypes and arise only years to decades after initiating carcinogens. However, there is still no coherent theory to explain these definitive characteristics of cancer. The prevailing mutation theory holds that cancers are late because the primary cell must accumulate 3–8 causative mutations to become carcinogenic and that mutations, which induce chromosomal instability (CIN), generate the individual karyotypes of cancers. However, since there is still no proven set of mutations that transforms a normal to a cancer cell, we have recently advanced the theory that carcinogenesis is a form of speciation. This theory predicts carcinogens initiate cancer by inducing aneuploidy, which automatically unbalances thousands of genes and thus catalyzes chain-reactions of progressive aneuploidizations. Over time, these aneuploidizations have two endpoints, either non-viable karyotypes or very rarely karyotypes of new autonomous and immortal cancers. Cancer karyotypes are immortalized despite destabilizing congenital aneuploidy by clonal selections for autonomy—similar to those of conventional species. This theory predicts that the very low probability of converting the karyotype of a normal cell to that of a new autonomous cancer species by random aneuploidizations is the reason for the karyotypic individuality of new cancers and for the long latencies from carcinogens to cancers. In testing this theory, we observed: (1) Addition of mutagenic and non-mutagenic carcinogens to normal human and rat cells generated progressive aneuploidizations months before neoplastic transformation. (2) Sub-cloning of a neoplastic rat clone revealed heritable individual karyotypes, rather than the non-heritable karyotypes predicted by the CIN theory. (3) Analyses of neoplastic and preneoplastic karyotypes unexpectedly identified karyotypes with sets of 3–12 new marker chromosomes without detectable intermediates, consistent with single-step origins. We conclude that the speciation theory explains logically the long latencies from carcinogen exposure and the individuality of cancers. In addition, the theory supports the single-step origins of cancers, because karyotypic autonomy is all-or-nothing. Accordingly, we propose that preneoplastic aneuploidy and clonal neoplastic karyotypes provide more reliable therapeutic indications than current analyses of *thousands* of mutations.

## 1. Introduction

In the words of John Maynard Keynes, “The difficulty lies not so much in developing new ideas as in escaping from old ones”.

Over a hundred years of research have shown that cancers have individual karyotypes [1,2,3,4,5,6] and arise only many months to decades after initiation by carcinogens [7,8,9,10,11,12,13,14]. However, there is still no coherent theory to explain these definitive characteristics of cancer. The prevailing mutation theory holds that cancers are slow to develop, because the primary cell must accumulate, stepwise, 3–8 specific gene mutations or chromosomes with cancer-specific gene mutations to become carcinogenic [15,16,17,18,19,20,21,22]. Further the mutation theory holds that the individual karyotypes of cancers are generated by mutations that induce *persistent* chromosomal instability (CIN) [23,24,25,26,27,28,29,30,31,32,33]. However, despite 65 years of research on the mutation theory, there is still no proof for even one set of mutations that is able to convert a normal cell to a cancer cell [15,16,29,34,35,36,37,38,39,40,41,42,43,44].

### Speciation Theory

Since the mutation theory continues to elude formal proof, we test here an alternative cancer theory. This theory holds that carcinogenesis is a form of speciation, because cancers share four definitive characteristics with conventional species [5,41,45,46,47,48], namely autonomy [49,50,51,52], karyotypic individuality [1,2,6,53], immortality [22,49,54,55] and the long latencies from carcinogens to cancers [5,11,13,41,56], which may be analogous to the long latencies from one conventional species to another [57,58,59,60,61]. 

**Figure 1:**
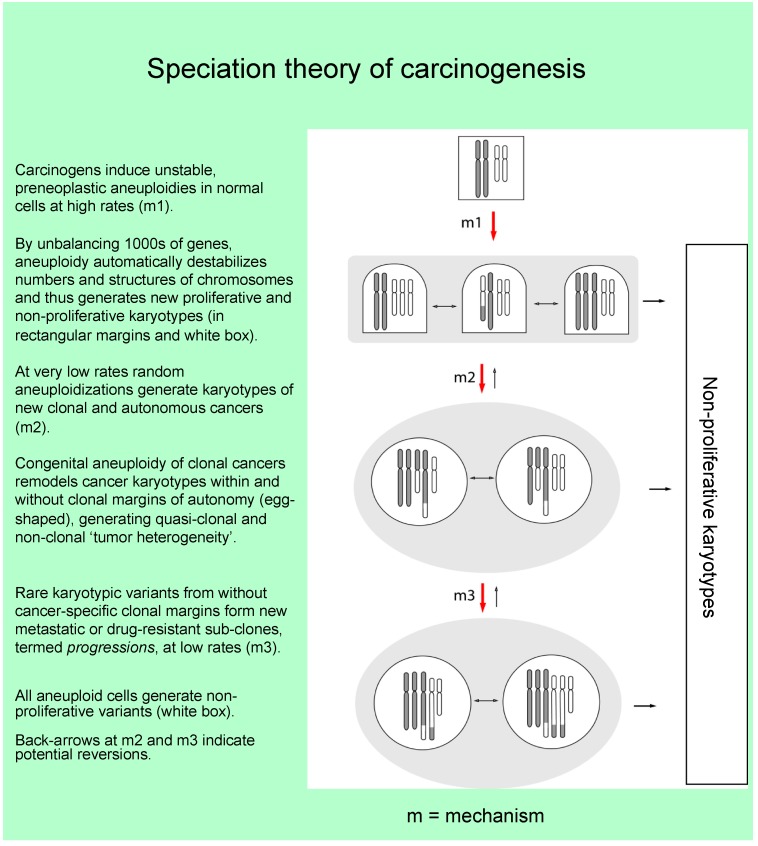
According to the speciation theory carcinogens initiate carcinogenesis by induction of aneuploidy. Aneuploidy destabilizes the numbers and structures of chromosomes and thus karyotypes automatically by unbalancing thousands of genes. Structurally rearranged hybrid or *marker* chromosomes are depicted by black and white bars. The resulting chain reactions of aneuploidizations then generate ever more aneuploid cells, which either form aneuploidy-dependent hyperplastic cells or more often non-proliferative cells (outside the gray rectangle in this graphic). Over time, these aneuploidizations have two endpoints, either non-proliferative karyotypes or very rarely karyotypes of new clonal cancer cells. Despite congenital aneuploidy, cancer karyotypes are stabilized against aneuploidy-catalyzed karyotypic degeneration by steady selections for cancer-specific autonomy and immortality. The resulting dynamic equilibrium between destabilizing aneuploidy and stabilizing selections for autonomy steadily remodels the karyotype generating quasi-clonal populations of cancer karyotypes, which oscillate between cancer-specific margins of variation (depicted as gray egg-shapes in this graphic). Owing to their inherent karyotypic flexibility, rare variants of cancer karyotypes stochastically form new sub-species with new phenotypes from without clonal margins of variations, such as metastasis and drug-resistance, which are termed progressions. The karyotypes of progressions are related to but distinct from parental karyotypes [75,76,77].

According to the speciation theory carcinogens initiate cancer by aneuploidization, which automatically unbalances thousands of genes and thus catalyzes chain reactions of progressive aneuploidizations [5,10,45,58,62,63,64,65,66,67,68,69,70]. Over time, these aneuploidizations have two endpoints, either non-viable karyotypes or very rarely karyotypes of a new autonomous cancer cell [5,55,71] (Figure 1). The low probability that random aneuploidizations generate a new autonomous cancer (or other species) explains why cancers have individual clonal karyotypes and are typically late [5,14,40,55,71,72,73,74,75]. The karyotypes of new autonomous cancer cells are stabilized and immortalized, despite destabilizing congenital aneuploidy, by clonal selection for autonomy and immortality [5,41] (Figure 1). The speciation theory would thus logically link the long preneoplastic aneuploidies with the typically rare and correspondingly late origins and individualities of cancers. This mechanism also predicts saltational, single-step origins, because autonomy is karyotypically all-or-nothing [5,41,71]—similar to conventional speciation [57,60,61].

Since cancer-specific aneuploidy (relative to normal precursor cells) automatically destabilizes cancer karyotypes by unbalancing previously homeostatic genes, cancer karyotypes are dynamic equilibria between destabilizing aneuploidy and stabilizing selections for cancer-specific autonomy. The resulting dynamic variations within cancer-specific clonal margins of autonomy generate the “clonal heterogeneity” or quasi-clonality of cancers [5,29,31,32,40,54,73,78,79,80,81,82,83,84,85] (Figure 1). 

This equilibrium of variation and stabilization of cancer karyotypes resolves a list of previously unexplained observations such as, (1) the paradox of “stability within instability” of cancers desrcibed by Gusev et al. [86], (2) “the remarkably stable genotypes” of transmissible cancers noted by Makino [87], Murgia et al. [51] and Murchison [88], (3) “the relative lack of evidence for further karyotypic evolution” of cancers noted by Wolman [89], (4) the co-existence of the facts that the “average (cancer) genotype is stable” despite “genome instability” and “substantial cell-to-cell variability” described by Albertson et al. [90], (5) the comments by Yoon et al. that “it is surprising that the modes of chromosomal numbers were remarkably consistent among different colonies of a given cell line, even when less than 50% of the cells within a colony had the model number of chromosomes…(and that) chromosomal counts apparently tended to return to a particular mode rather than drift toward increasing levels of aneuploidy” [91], and (6) the recent observation by Wangsa et al. that “most single cell-derived daughter lines (of cancers) maintained their major clonal pattern” [92]. 

The relatively high quasi-clonal and non-clonal flexibility of cancer karyotypes, compared to the karyotypes of normal species, also predicts the notorious progressions of cancer [93]. Progressions are rare sub-species of cancers with distinct new phenotypes, such as metastasis and drug-resistance, and with distinct karyotypes that are, however, related to those of parental cancers [75,76,77,94]. This origin of progressions from flexible cancer cells would explain why progressions of cancers—as cancers of cancers—occur more often than new cancers occur in stable normal cells (see Figure 1).

However, in the absence of a current textbook theory, karyotypic tumor heterogeneity is typically described as “chaotic” [22,95] or as “genetic noise” [95] or as “genome instability” [90] or as “neither a clonal marker nor an initial event” [96] or as CIN [23] (above). Yet according to a recent review “there is currently no single mechanistic explanation” for CIN, because “most chromosomally unstable cancer cell lines have functional spindle assembly checkpoints” [97].

In an effort to resolve current inconsistencies between the putative roles of clonal versus non-clonal karyotypes and the sources of individual cancer karyotypes, we test here experimentally the explanations of the speciation theory for the karyotypic individuality of cancers and for the long latencies from carcinogens to cancers. 

## 2. Materials and Methods 

### 2.1. Cell Cultures

Rat lung primary cells were prepared from the lung of a young adult (<1 year-old) Sprague Dawley rat from the Office of Laboratory Animal Care (University of California at Berkeley) following published procedures [14]. The neoplastic F10 rat clone was derived from a culture of SV40 virus-infected rat lung cells as described by us previously [14]. Primary human mesothelial cells were prepared and infected with SV40 virus as described by Bocchetta et al. [98]. The cells were grown on plastic culture dishes (Corning Falcon, NY, USA) in RPMI 1640 medium (Corning Cellgro, MA, USA) supplemented with 3–5% fetal calf serum, 3–5% calf serum (Hyclone, UT, USA), 1% *Antibiotic Antimycotic* and 1% *Nyastin* (Sigma Co., St Louis, MI, USA) following published procedures [14,71].

### 2.2 Karyotyping of Rat and Human Cells by Hybridization of Metaphase-Chromosomes with Color-Coded Chromosome-Specific DNA Probes

To optimize the percentages of cells in metaphase, appropriate cultures were seeded one to two days before karyotyping, at about 50% cellular confluence in a 5-cm or 10-cm culture dish with 3 mL or 6 mL of the medium described above. After reaching ~75% confluence, about 300 ng colcemid was added to 3 mL medium, which corresponded to 30 µL of a commercial *KaryoMax* solution at 10 µg/mL (Gibco Inc., Waltham, MA, USA). The culture was then incubated at 37 °C for 4–8 h. Subsequently, cells were washed with 3 mL of physiological phosphate buffered saline and then suspended in 0.075 M KCl and incubated at 37 °C for 15 min. The cell suspension was then cooled in ice-water, mixed (or *prefixed*) with 0.1 volume of the freshly mixed glacial acetic acid-methanol (1:3, vol. per vol.) and centrifuged at 800 g for 6 min at room temperature. The cells were then pelleted and suspended in about 100 μL supernatant and mixed drop-wise with 5 mL of the ice-cold acetic acid-methanol solution and then incubated or fixed at room temperature for 15–30 min or at 5 °C overnight. The cells of this suspension were then pelleted and either re-suspended in fixative and pelleted once more or were directly pipetted on a microscope slide for microscopic examination of metaphases at 200-fold magnification with phase-contrast optic. Under these conditions, metaphase chromosomes attach to glass slides as the solvent evaporates. Slides with suitable metaphases are then identified under the phase-contrast microscope and hybridized to color-coded, rat or human chromosome-specific DNA probes as described by the manufacturer, MetaSystems (Newton, MA, USA). Color-coded chromosomes of metaphases were then sorted into conventional karyotypes with a computerized Zeiss Imager M1 microscope (Zeiss, Jena, Germany) programmed by MetaSystems, following published procedures [14,71]. 

## 3. Results

In the following we describe experimental evidence for the predictions of the speciation theory that cancers are generated by rare phylogenetic rearrangements of the karyotypes of normal cells—similar to those thought to have generated new phylogenetic species [59,60,61,99]. According to the speciation theory of carcinogenesis, the karyotypic individuality of cancers and the long latencies from carcinogens to cancer are thus direct consequences of the low probability of generating a new autonomous species by random karyotypic rearrangements (Figure 1).

### 3.1. Initiation of Carcinogenesis by Induction of Preneoplastic Aneuploidy

The speciation theory holds that the first step in carcinogenesis is induction of preneoplastic aneuploidy by mutagenic or non-mutagenic carcinogens. Once established, aneuploidy initiates autocatalytic progressions of aneuploidy [5,41,67] (Figure 1). To test this prediction, we analyzed the karyotypes of mammary tissues of BUF/N rats (National Cancer Institute, Frederick, MD, USA) several months after injection with a single carcinogenic dose of mutagenic methyl-nitrosurea (NMU), but prior to carcinomagenesis as described by Aldaz et al. [100]. Aldaz et al. provided these preneoplastic rat tissues to us for a collaborative study of chemical carcinogenesis in 2014 [74] and for further studies. In addition, we analyzed the karyotypes of cultured primary human mesothelial cells at various months after infection with SV40 tumor virus and again months before neoplastic transformation, as described by Bocchetta et al. [98]. The SV40-infected mesothelial cultures studied here were kindly prepared and infected with SV40 by Michele Carbone and Fang Qi from the Cancer Center of the University of Hawaii at Honolulu.

#### 3.1.1. Preneoplastic Aneuploidy in Rats Injected with Nitrosourea 

To test for induction of preneoplastic aneuploidy by a mutagenic carcinogen, we investigated the karyotypes of preneoplastic rat mammary hyperplasias prepared between two and three months after injection with nitrosourea, as described above. Under these conditions, the rats tested only generate mammary carcinomas at least three months after injection [100]. 

For the detection of aneuploidy prior to carcinogenesis, we compared karyotypic patterns formed by about 20 individual cells of preneoplastic rat mammary tissue to karyotypes of normal rat lung tissue (Materials and Methods) using karyotype-arrays. Such karyotype-arrays were developed by us recently to detect cancer-specific karyotypic clonality in the presence of concurrent non-clonal neoplastic and preneoplastic karyotypes [5,73,74,81]. 

**Figure 2:**
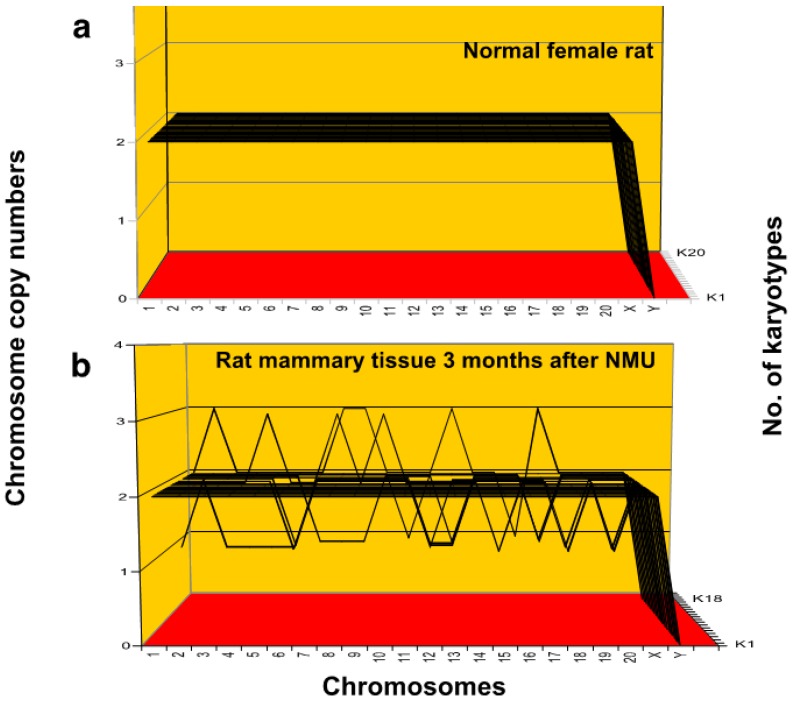
Karyotype-arrays of (**a**) normal female rat and of (**b**) preneoplastic rat mammary tissue 3 months after methyl-nitrosourea (NMU). Karyotype-arrays compare the copy numbers of the chromosomes of multiple cells in three-dimensional diagrams. Such diagrams list the chromosome numbers of individual karyotypes on the *x*-axis, the copy numbers of each chromosome on the *y*-axis, and the number of karyotypes arrayed on the *z*-axis as described previously by us [14,71] and others [102]. The preneoplastic rat mammary tissue was derived from hyperplastic rat mammary tissue two to three months after subcutaneous injection with 50–100 mg NMU as described in the text. The origin of normal rat tissue is described in Materials and Methods.

As can be seen in Figure 2a,b, karyotype-arrays list the chromosome numbers on the *x*-axis, the chromosome copy numbers on the *y*-axis and the numbers of the individual karyotypes compared on the *z*-axis [5,73,81]. As expected, the karyotype-array of 20 normal female rat lung cells, shown in Figure 2a, formed 20 completely parallel lines, indicating karyotypes with identical chromosome copy numbers, namely 2. This array thus shows at a glance that all 20 cells of the normal rat tested were diploid. 

In contrast, Figure 2b shows that 9 of 18 (50%) preneoplastic cells of rat mammary tissue prepared two to three months after treatment with nitrosourea were randomly aneuploid. As can be seen in Table 1, three of the nine aneuploid cells contained one aneuploid chromosome and six contained multiple aneuploid chromosomes with non-diploid copy numbers. Moreover, it was shown by us previously that all cancers resulting from these and all similar preneoplastic tissues tested occurred late and contained new individual clonal karyotypes [14] (Figure 1). 

In view of this, we conclude that the early random and non-clonal aneuploidies of nitrosourea-treated rat mammary cells analyzed here are direct precursors of the late, individual and clonal karyotypes of the resulting cancers described by us previously [14,74] (see also Figure 1). 

A study by Goepfert et al. using the same system as Aldaz even showed aneuploidy within a day after inoculation of the rats with nitroso-urea [101]. This virtually excludes an intermediate mechanism between carcinogen and aneuploidization. Consistently, the long latent periods from carcinogen to cancers in this system depend on the low probability of forming carcinogenic karyotypes from random preneoplastic aneuploidies—as predicted by the speciation theory.

The conclusion that preneoplastic aneuploidy is necessary for karyotypic carcinogenesis is further supported by earlier observations that preneoplastic aneuploidies of carcinogen-treated Chinese hamsters, mice and rats consistently segregated with subsequent clonal cancer-specific aneuploidies [14,74,101,103,104]. 

By contrast, mutated *ras* genes, which were originally thought to be the proximate causes of transformation of rat tissues injected with NMU, were found in only 50% of the rat carcinomas generated by nitrosourea—indicating that these mutations were not necessary for carcinogenesis [100].

#### 3.1.2. Preneoplastic Aneuploidy in Human Mesothelial Cells Initiated with SV40 Tumor Virus

In a parallel experiment, we searched for preneoplastic aneuploidy in normal human mesothelial cells at various times after initiation of carcinogenesis by infection with SV40 virus. As described above, Michele Carbone and Fang Qi from the Cancer Center of the University of Hawaii at Honolulu kindly prepared these cells for our study.

In contrast to nitroso urea, SV40 is a non-mutagenic biological carcinogen, which destabilizes karyotypes with aneuploidogenic viral proteins indefinitely [14,64,65]. At low rates SV40 can also function as mutagenic carcinogen by integrating SV40 DNA in cellular chromosomes [14,105].

**Figure 3:**
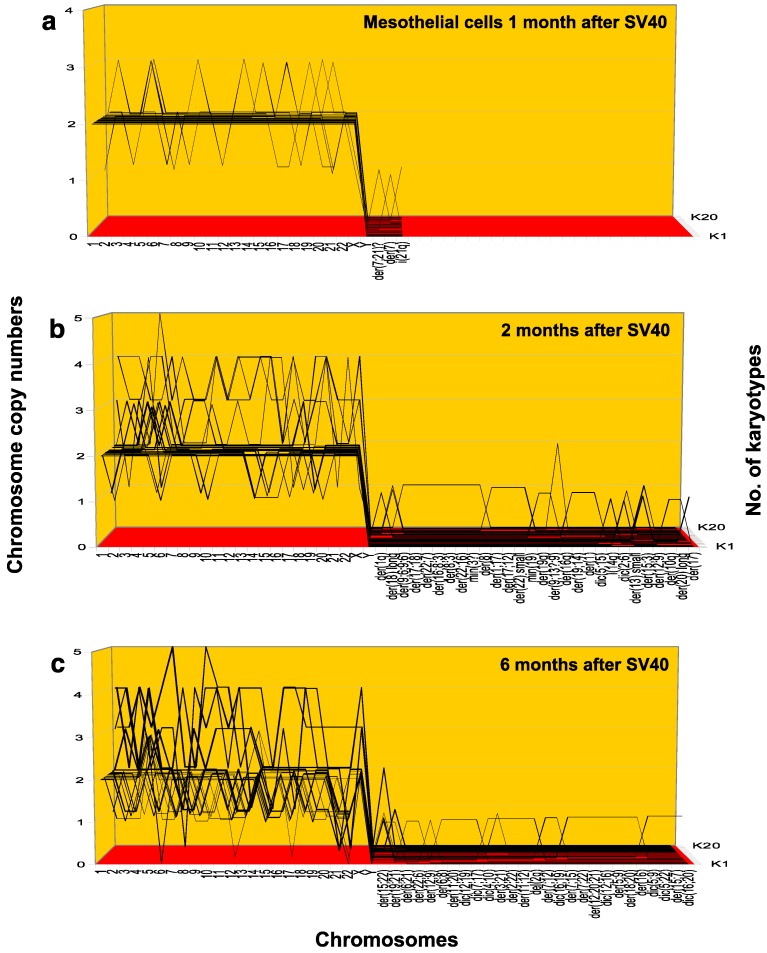
Karyotype-arrays of mesothelial cells at (**a**) one, (**b**) two and (**c**) six months after infection with oncogenic SV40 virus. As described for Figure 2, karyotype arrays are three-dimensional tables that list chromosome numbers on the *x*-axis, copy numbers on the *y*-axis and the number of karyotypes compared on the *z*-axis. Quantitative lists of the copy numbers of all intact and marker chromosomes of the three arrays are shown in Table 2a–c. The three arrays show the autocatalyzed increase of aneuploidization over time predicted by the speciation theory (Figure 1). The steady increase of preneoplastic aneuploidy after initiation of carcinogenesis by SV40 shown here, confirms the time-dependence of exponential preneoplastic aneuploidization prior to carcinogenesis. Marker chromosomes are as described and defined in Table 2. Accordingly, der stands for derivative and dic for dicentric chromosomes.

To test for the predicted time-dependent progression of aneuploidy in SV40-infected normal human mesothelial cells (Figure 1), we sampled karyotypes of infected cultures at zero, one, two and six months after infection. As expected, at zero time 20 normal mesothelial cells were all diploid, forming a karyotype-array of 20 parallel lines (as shown above in Figure 2a for normal rat cells, but not here for human cells). By contrast, we show in Figure 3a and the corresponding Table 2a that one month after infection with SV40, 13 of 20 (65%) mesothelial cells were aneuploid (yellow highlights in Table 2a). In addition, 3 of the 13 aneuploid cells had also each acquired distinct marker chromosomes, which are also shown in Table 2a.

**Figure 4:**
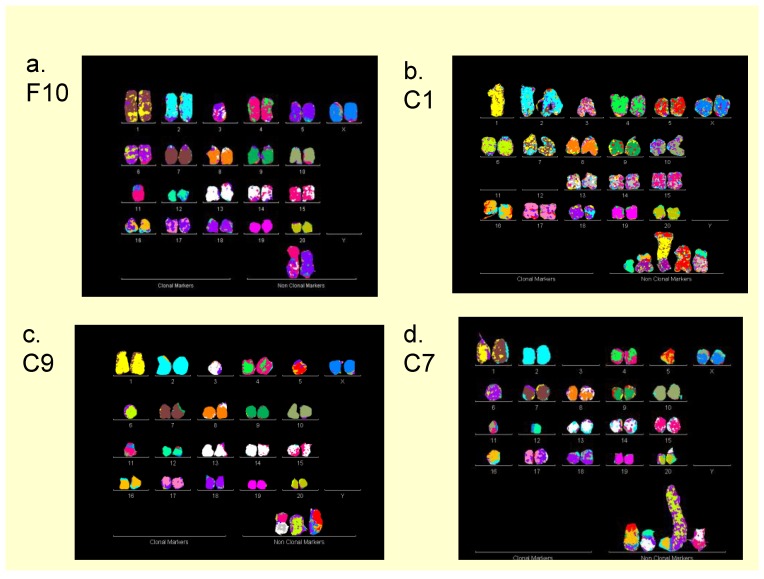
Metaphases of color-coded chromosomes of (**a**) the neoplastic rat clone F10 and of three F10-sub-clones (**b**) C1, (**c**) C7 and (**d**) C9. To test the heritability of the karyotype of the SV40-induced neoplastic rat clone, metaphase chromosomes of the parental F10 and of three single-cell-derived subclones C1, C9, and C7 were compared. As can be seen by comparisons of their metaphases the four karyotypes were closely related, but not identical. For example, the copy numbers of the disomic parental chromosome (Chr) 1 were 75% clonal, those of the parental disomic Chr 2 were 100% clonal, those of the parental monosomic Chr 3 were 75% clonal, those of the parental disomic Chr 4 were 100% clonal, those of the parental disomic Chr 5 were 50% clonal and those of the parental disomic Chr 6 were 100% clonal, etc. In addition, there were also 2–5 sub-clone-specific marker chromosomes per metaphase that are hybrids of two to three intact chromosomes and carry parts of the missing intact chromosomes. In order to obtain more quantitative estimates of heritability, we performed the comparisons of 20 karyotypes of the parental clone and of six sub-clones based on the karyotype arrays shown in Figure 5a–g. The compositions of the marker chromosomes in Figure 4a–d are listed in karyotypes #6 (Table 3a), #5 (Table 3b), #9 (Table 3g), and #1 (Table 3e), respectively.

Notably, marker chromosomes are hybrids formed of two or more different chromosomes as well as by intra-chromosomal rearrangements, which are all catalyzed by aneuploidy, just like numerical chromosome variations [14,72,75,106] (Figure 1). See also examples of marker chromosomes in a neoplastic rat clone, shown below in Figure 4.

Consistent with the predicted autocatalytic progression of aneuploidy, the aneuploidy of SV40-infected mesothelial cells had increased from 65% at one month to 95% at two months. At the same time, the number of new marker chromosomes per 20 cells had increased exponentially from three at one month to 28 at two months. This result confirmed and extended an earlier test of preneoplastic aneuploidy in mesothelial cells infected with SV40 [14,74].

At six months after initiation of carcinogenesis with SV40, 100% of the mesothelial cells had become aneuploid as shown in Figure 3c and the corresponding Table 2c. In parallel with the increasing degrees of aneuploidy of intact chromosomes, the numbers of new marker chromosomes per 20 cells had again increased exponentially with time after infection to 112 compared to only 3 at one month and 28 at three months after infection, as can be seen in Figure 3a–c and in Table 2a–c. 

It can also be seen in Figure 3c and Table 2c that there is evidence for emerging karyotypic clonality six months after infection by SV40, namely parallel lines formed by identically abnormal chromosome copy numbers of several chromosomes among different mesothelial cells. Karyotypic clonality late after initiation of carcinogenesis is also consistent with the late origins of neoplastic clones, which we as well as others have described previously [14,74,98].

Based on these findings and on earlier observations of induction of preneoplastic aneuploidy with non-mutagenic carcinogens listed in the Introduction [5,10,45,58,62,63,64,65,66,67,68,69,70], we deduce that mutagenic and non-mutagenic carcinogens initiate carcinogenesis by preneoplastic aneuploidization, independent of gene mutations. 

Collectively, these findings demonstrate that carcinogen-induced aneuploidy precedes malignant transformation in rats treated with mutagenic nitroso-urea and in human cells treated with non-mutagenic SV40 virus. This is apparently also true in all other systems of experimental carcinogenesis tested by us [5,74]. Preneoplastic aneuploidy is thus a testable proximate cause for the formation of new cancer-specific karyotypes. Consequently, the low probability of forming new phylogenetic cancer karyotypes by random aneuploidizations stands out as the only consistent explanation for the long latencies of carcinogenesis and the subsequent formation of individual clonal cancer karyotypes (see Figure 1).

By contrast there is still no testable evidence for preneoplastic and neoplastic sets of cancer-causing gene mutations. 

### 3.2. Preneoplastic Karyotypes with Multiple New Marker Chromosomes—Evidence for Saltational Origins 

Unexpectedly, our quantitative analyses of the karyotypes of SV40-infected, preneoplastic mesothelial cells revealed karyotypes with multiple new markers, ranging from 3 to 12 per karyotype, in the absence of intermediates. In other words, there were no intermediate karyotypes with fewer markers than in these sets of 3–12 new markers (Table 2b,c).

This result was unexpected, because the prevailing theory holds that cancer karyotypes are generated from normal karyotypes by stepwise or gradual accumulation of new cancer-specific chromosomes [17,18,22]. However, since the karyotypic intermediates predicted by the gradual accumulation theory were never found, several researchers [107,108] including us [71,77] have recently advanced the theory that cancer karyotypes arise from random preneoplastic aneuploidies in single steps. Consequently, this view would predict karyotypic origins of cancers without intermediates—analogous to the saltational chromosomal rearrangements thought to have generated conventional species [57,58,59,60,61]. If correct, this result would support the speciation theory, because karyotypes of autonomous species are all-or-nothing. In other words, intermediates would not be stabilized by selections for autonomy.

In view of this, we analyzed the composition and frequency of the preneoplastic karyotypes with 3–11 new marker chromosomes with the following results:

Starting with human mesothelial cells two months after infection with SV40 (Figure 3b), we found that 1 in 20 cells contained three, another four, and a third cell even contained eight distinct new marker chromosomes (seen as non-clonal lines in the right half of Figure 3b and marked yellow in Table 2b). None of these new marker chromosomes were found in any other of the 20 cells analyzed.

Furthermore, Figure 3c and Table 2c show that six months after infection with SV40 even more sets of multiple new marker chromosomes were present in 20 cells than two months after infection, namely 2 karyotypes with 4, 2 with 5, 2 with 6, 1 with 7, 1 with 8, 2 with 9, 1 with 10, 1 with 11 and 1 with 12 new individual marker chromosomes. There were again no other cells with these new marker chromosomes in any of the 20 pre-neoplastic mesothelial cells six months after infection with SV40 (Figure 3c and Table 2c). Moreover, none of these new markers were found in mesothelial cells one month and two months after SV40 infection (Table 2a,b). These results suggest that most, perhaps all new preneoplastic karyotypes with multiple new marker chromosomes are assembled saltationally, in single steps.

### 3.3. Individual Karyotypes of Cancers Are Heritable and Thus Segregate with All Sub-Clones 

The speciation theory predicts that cancers are generated and maintained by heritable clonal karyotypes—similar to conventional species.

The predicted heritable clonal cancer karyotypes are, however, often overlooked as potential causes of cancers, because the clonality of cancer karyotypes is masked by heterogeneity within and without clonal margins of autonomy, which are much wider than those of conventional species (see Introduction and Figure 1). As a result of this “heterogeneity”, cancer karyotypes are widely interpreted as consequences of cancer-specific chromosome instability or CIN mutations [23,24,25,26,27,28,29,30,31,32,33]. According to this CIN-theory cancers have individual and unstable, passenger karyotypes, which would not be heritable.

To distinguish between the heritable clonal karyotypes predicted by the speciation theory and the non-heritable, unstable passenger karyotypes predicted by the CIN-theory, we examined the heritability of individual cancer karyotypes in sub-clones of cancers, derived from single cancer cells. Under these conditions heritable karyotypes would segregate with all sub-clones, whereas unstable, individual CIN-induced karyotypes would not, or would not consistently, segregate with sub-clones.

For this purpose, we compared the karyotype of the neoplastic rat clone F10 with the karyotypes of six single-cell-derived sub-clones of F10. The neoplastic F10 clone was derived from an SV40-infected culture of primary rat lung cells as described previously [14] and in Materials and Methods. The six single cell-derived sub-clones were prepared by classic endpoint dilutions of F10 and were termed C1, C2, C6, C7, C8 and C9, respectively.

As a preliminary test for heritable karyotypes, we compared the chromosomes of metaphases of the parental F10 clone and of three F10-subclones, C1, C7 and C9, as shown in Figure 4a–d. This comparison revealed the following percentages of clonal heritability in terms of chromosome copy numbers among the four karyotypes: disomy of chromosome (Chr) 1 = 75%; disomy Chr 2 = 100%; monosomy Chr 3 = 75%; disomy Chr 4 = 100%; disomy Chr 5 = 50%; disomy Chr 6 = 100%; etc. Notably, actual percentages of heritability of chromosomes with less than 100%, e.g., chromosomes 1, 3, 5, etc., are in fact higher than the percentage of missing intact chromosomes indicates, because sub-clonal marker chromosomes carry parts of the missing intact chromosomes, as is shown on the bottom lines of the metaphases shown in Figure 4 and below in Table 3. It would appear then that the karyotypes of F10 are more or less heritable in the three sub-clones C1, C7 and C9.

**Figure 5:**
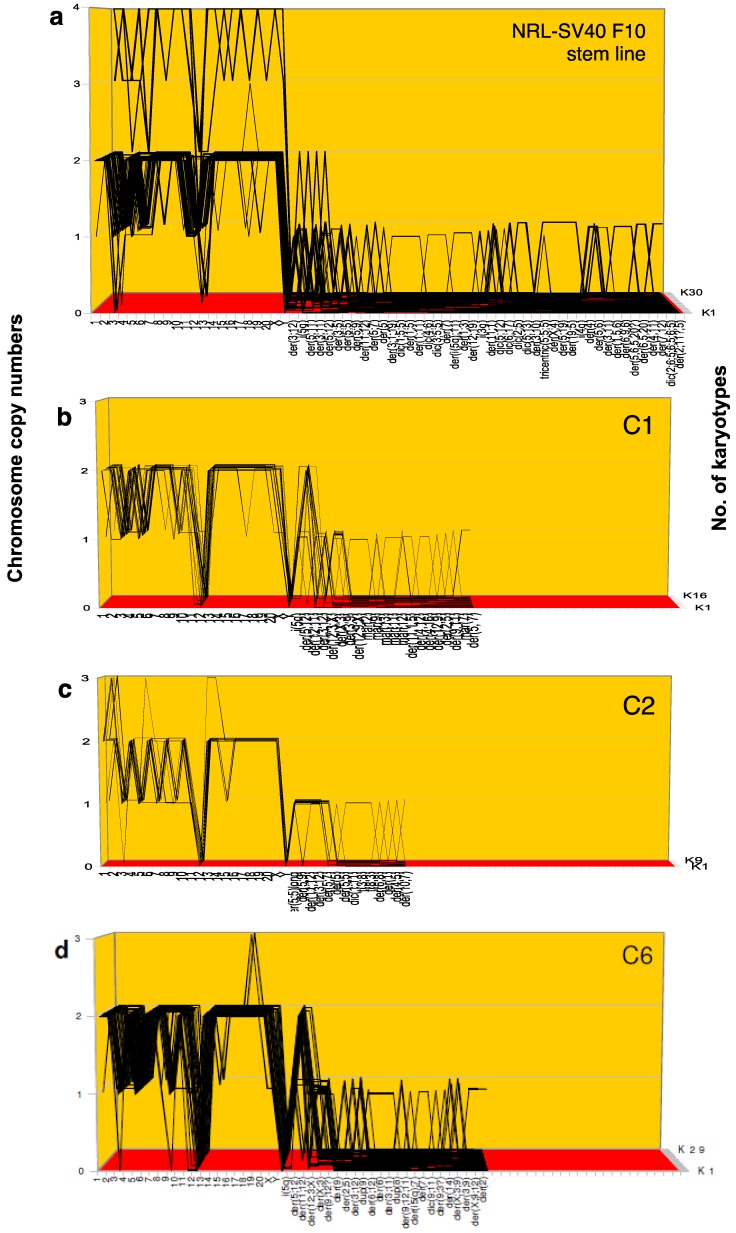

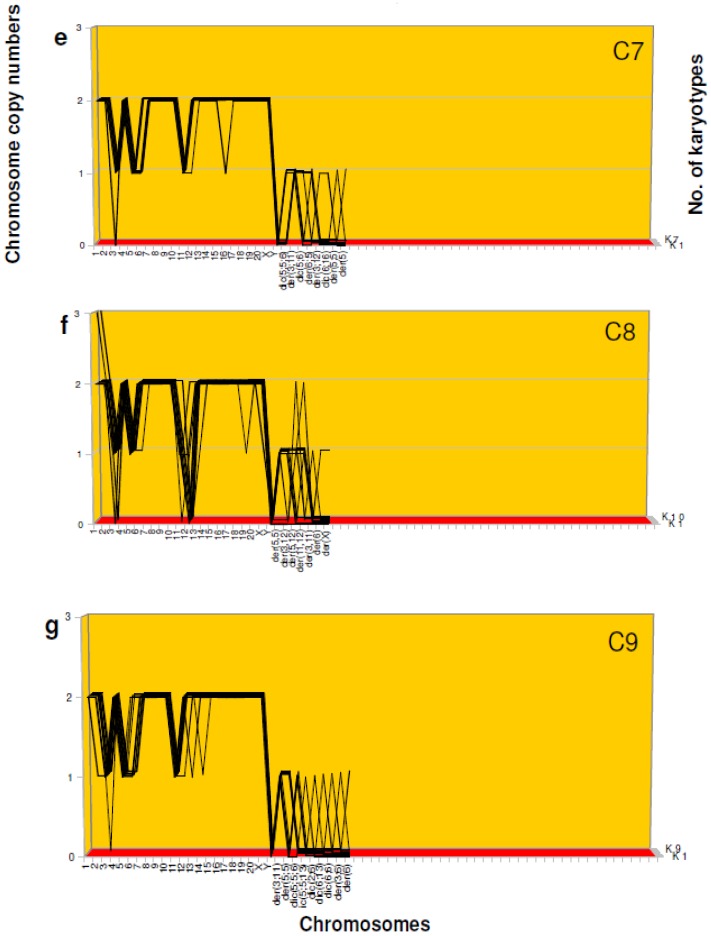
A comparison of the chromosome copy numbers and structures of the neoplastic rat clone F10 and six individual sub-clones. This comparison was undertaken to determine whether the karyotypes of cancers are clonally heritable, as predicted by the speciation theory, or are unstable and thus non-heritable as predicted by the CIN-theory, which predicts that cancer karyotypes are non-heritable consequences of persistently acting, cancer-specific mutations that cause chromosome instability (CIN) (see Introduction and References [23,24,27,28,29,30,33]). As can be seen in the karyotype-arrays of the primary F10 clone (**a**); and of the six sub-clones C1, C2, C6, C7, C8 and C9 (**b**–**g**), the parental clone and its six sub-clones have closely related karyotypes (see also Table 3 and Table 4). For example, the primary F10 clone and its sub-clones share identical or closely related, ±1 copy numbers of intact chromosomes and several sub-clonal marker chromosomes. Moreover, new clonal marker chromosomes containing parts of the missing intact chromosome compensate for several sub-clonal losses of intact clonal chromosomes. For example, the monosomy of chromosome 7 in C2 is compensated by a clonal marker chromosome der (5,7). This close karyotypic relationship between primary cancer and six independent sub-clones is consistent with a heritable quasi-clonal cancer karyotype.

In an effort to obtain statistically further evidence for the heritability of cancer karyotypes, we compared karyotype-arrays of the paternal F10 clone with arrays of each of the six sub-clones C1, C2, C6, C7, C8, C9, which is the method described above in Figure 2 and Figure 3. As can be seen in Figure 5a–g, the corresponding Table 3a–g and specifically in the overview presented in Table 4, the karyotype-arrays of the F10 clone and of all six sub-clones are about 70–90%-related. This indicates that the copy numbers of the chromosomes of F10 are quasi-stable and heritable—“rather than drift(ing) toward increasing levels of aneuploidy” as observed previously by Yoon et al. [91].

In the following we show first a synopsis of the karyotype arrays of the neoplastic F10 rat clone next to the arrays of the six single-cell-derived sub-clones mentioned above. This review of the seven arrays is followed by seven corresponding tables listing the constituent chromosomes of the individual karyotypes of F10 and its sub-clones in Table 3a–g. The essential evidence for chromosomal and karyotypic heritability is then summarized in Table 4.

As shown in Table 4, the following two elements of heritable chromosomal clonalities were found in Figure 5 and Table 3: The average number of chromosomes per F10 karyotype was 45.1 and those of the six F10 sub-clones varied between 40.3 and 42.7. Specifically, the average clonalities of the predominant copy numbers of all intact chromosomes of F10 and the six sub-clones were found to range in percent between 83 (F10), 89 (C1), 87 (C2), 90 (C6), 96 (C 7), 94 (C8) and 96 (C9).

These percentages were calculated based on the sum of all intact chromosomes of each kind per clone and are listed in Table 4. In addition, a few clone-specific chromosome copy number variations in the narrow range of ± 1 were observed (see Figure 5a–g and Table 4), as predicted by the speciation theory. This narrow range of chromosome number variation reflects the equilibrium between constitutive karyotypic destabilization by congenital aneuploidy and the concurrent selections for autonomy (Figure 1).

Table 4 also lists clonalities of marker chromosomes of individual clones as recorded in Table 3a–g. The clonalities of markers fall into two groups, (1) those that are highly, 75–100%, clonal and are shared in five cases between two or three sub-clones, and (2) those that are sub-clonal or non-clonal that are also shown in Table 3 and Table 4. For example, Table 4 shows that several F10 sub-clones and F10 share highly clonal sub-clone-specific marker chromosomes including C1, C2 and C8; C7 and C9; C1, C6 and C8; and C1, C2 and C6. By contrast, most karyotypes with non-clonal and some with sub-clonal marker chromosomes, shown in Figure 5 and Table 3, are apparently unique and rarely achieve clonal viability.

In sum co-segregation of the quasi-clonal chromosome numbers and of some F10-specific sub-clonal marker chromosomes of the neoplastic F10 clone with its sub-clones supports the theory that cancer karyotypes are heritable and genomic.

On the other hand, there is also evidence for some heritable variability among the related rat clones from within and without the clonal margins of autonomy and immortality (Figure 1), which is consistent with aneuploidy-catalyzed variation of cancer karyotypes. For example, Table 3 and Table 4 show that each F10 sub-clone contained some individual non-clonal or sub-clonal marker chromosomes that were not found in other sub-clones.

Thus, we can now offer experimental evidence for heritable genomic cancer karyotypes, to explain the individuality of the karyotypes of cancers. Contrariwise chromosomal instability caused by CIN mutations would have predicted predominantly sub-clones with unpredictable and unstable karyotypes [23,24,25,26,27,28,29,30,31,32,33]. In addition, persistent generation of non-clonal chromosome instability by CIN would be inevitably lethal in terms of Muller’s ratchet [55,109] and would thus not be compatible with the definitive immortality of cancers [22,54,55].

In the following, we briefly address the question of how the complex individual karyotypes of cancers are assembled from parental chromosomes.

### 3.4. Saltational Origins of Neoplastic Karyotypes 

The speciation theory favors saltational origins of cancer karyotypes. This is because karyotypic intermediates would not be stabilized by selections for karyotypic autonomy [71,77], since karyotypes of autonomous species are “all-or-nothing” [57,60,61]. 

Unexpectedly, in view of the multi-step mechanism advanced by the mutation theory, we found in our quantitative analyses of the karyotypes of the neoplastic clone F10 and its sub-clones several otherwise clonal karyotypes with 3–5 new, individual marker chromosomes (yellow highlights in Table 3)—without any detectable karyotypic intermediates (containing sub-sets of these multiple marker chromosomes). The following F10-related karyotypes with multiple new marker chromosomes were found in the karyotype arrays shown in Figure 5 and Table 3a–g:

(1) Figure 5a and Table 3a show, among 30 F10-karyotypes, four karyotypes with unique sets of 3–5 new marker chromosomes (single graphic lines in Figure 5a), which were not found in the remaining 29 karyotypes.
(2) Figure 5b and Table 3b show, among 16 C1-karyotypes, one karyotype with three new marker chromosomes, which were not found in the remaining 15 C1 karyotypes.
(3) Figure 5c and Table 3c show, among nine C2-karyotypes, one karyotype with four new marker chromosomes, which were not found in the remaining 8 C2 karyotypes.
(4) Finally Figure 5d and Table 3d show, among 29 C6-karyotypes, two karyotypes with three new marker chromosomes, which were not found in the remaining 28 karyotypes.

Thus, there were eight karyotypes with 3–5 new individual marker chromosomes among a total of 82 karyotypes of F10 and its sub-clones. In other words, there were no hypothetical “step-wise precursors” [17,18] or intermediates of these nine karyotypes in any of the 82 karyotypes of F10 and the six F10-sub-clones we analyzed. Furthermore, we did not even include here the cases of karyotypes with two unique marker chromosomes without precursors, which are listed in Table 3a–g.

In view of this, it is hard to avoid the conclusion that the unique sets of 3–5 new marker chromosomes associated with eight variants of F10-karyotypes were assembled simultaneously in single steps, as we [71,77] and others [107,108] proposed recently for other cancer cells.

It could be argued, however, that intermediates were not found, because they are not autonomous and are thus too short-lived for detection. However, this argument fails to explain how such short-lived intermediates could cause natural cancers only after the typically long latencies of cancers of years to decades (Introduction).

Moreover, the absence of intermediates among short-lived preneoplastic karyotypes with up to 12 new marker chromosomes virtually excludes a gradual chronological accumulation of new marker chromosomes (see Figure 3 in Section 3). Statistically, at least some precursors with fewer markers should have been found.

Taken together, these results support the prediction of the speciation theory that neoplastic cancer karyotypes with multiple new marker chromosomes are assembled in single steps—analogous to the saltational origins of conventional species [57,60,61].

## 4. Conclusions

### 4.1. The Long Latent Periods and Individual Karyotypes of Cancers in the Light of the Speciation Theory

Our experiments on the speciation theory of cancer provide a coherent explanation for two previously unexplained characteristics of carcinogenesis—the karyotypic individuality and the long latent periods of cancers, as follows:

(1) *Long latent periods from initiating carcinogens*. We adduced verifiable evidence that the low probability of creating karyotypes of new autonomous cancer species by random aneuploidizations of the karyotypes of precursors, is the reason for the long latencies of carcinogenesis.
(2) *Heritable individual karyotypes of cancers*. Challenged by the prevailing theory that the individual karyotypes of cancers are non-clonal products of CIN mutations and thus not heritable genomes, we found instead that the karyotypes of cancers are quasi-clonal and heritable—similar to those of conventional sexual species. (Nevertheless, the karyotypes of sexual species are much less variable than those of cancers [97,110].) The experimental evidence of this finding was that the quasi-clonal karyotype of the neoplastic rat clone F10 segregated with six out of six single cell-derived sub-clones with only minor sub-clonal variations.

It follows that the quasi-clonal karyotypes of individual cancers are the heritable genomes of cancer-species. This conclusion would also explain the ubiquity of individual karyotypes in all 68,000 cancers reported by the NCI-Mitelman database [6].

(3) *Evidence for single-step origins of cancer karyotypes*. Unexpectedly, in view of the multi-step theory of carcinogenesis, we found sub-clones of F10 karyotypes with individual sets of 3–5 new marker chromosomes, but no detectable precursors carrying subsets of these new markers. We thus concluded that these unique sets of marker chromosomes were assembled in single-steps, rather than gradually by a stepwise mechanism. The suspected single-step origin of cancer karyotypes is further supported by our findings of karyotypes with 3–11 new marker chromosomes without precursors in aneuploid preneoplastic mesothelial cells (Table 2b,c).

These results confirm and extend prior evidence, including ours, for single-step carcinogenesis in human cancers [77,107,108] and in experimental mice [71]. Therefore, we conclude that the new and previous evidence for the creation of new cancer species in single steps lends independent support to the speciation theory. 

The rather simple chromosomal explanations for the *karyotypic individuality* and *long latency* of cancers have probably been overlooked previously for conceptual and technical reasons:

*Conceptual reason*. Because the prevailing mutation theory has dominated the search for the genetic causes of cancer since the discovery of gene mutation in 1927 [111], consistent mutations or consistent karyotypic abnormalities with specific mutations were expected [15,21,22]. Instead, individual mutations [43,44] and individual karyotypes [2,9,112,113] were found, of which over 68,000 are listed in the NCI-Mitelman database of cancers [6]. Moreover, genetic individualities of cancers were disregarded, despite evidence for phenotypic individualities of caner of the same type [5,18,19,44,95]. In sum genetic and phenotypic individualities were overlooked, because of the absence of a coherent theory for individualities. In the words of the late UC Berkeley professor Gunther Stent “The best data are useless, if they cannot be confirmed by theory”. 

*Technical reason.* For technical reasons the quasi-clonal, individual karyotypes of cancers have been disregarded or overlooked as candidates of causation, because of their inherent “heterogeneity” [9,80,95,112,114,115]. 

Owing to this heterogeneity, the karyotypic clonality of cancers is typically obscured in conventional cytogenetic analyses by concurrent heterogeneity and thus not recognized as the logical cause of cancers [95,112,116]. As a result, cancer karyotypes are typically described as “chaotic” [22,95], or as “cytogenetic noise” [95], or as consequences of “persistent” CIN [23], or as “neither a clonal marker nor an initial event” [96] (See also Introduction). It is for this reason that textbooks and leading articles use non-causal surrogate markers as evidence for the clonality of cancers, such as clonal maternal or paternal X-chromosomes [22,117,118]. 

The technical problems of detecting the clonality of cancer karyotypes in the presence of heterogeneity were solved here by the use of karyotype-arrays. As shown in Figure 5, the karyotype-arrays of neoplastic rat clones provided direct evidence for our conclusion that cancers are individual species with quasi-clonal, heritable karyotypes of their own.

### 4.2. Clinical Relevance of Heritable Individual Karyotypes and Preneoplastic Aneuploidies of Cancers

Most current efforts to understand and treat cancers are focused on gene mutations, although there is still no consistent correlation between cancers and any specific set of the ever-growing numbers of gene mutations of cancers found by whole genome sequencing [42,43,44,119,120]. However, despite the “thousands of exacerbating intratumor mutations” currently diagnosed [29] and “despite the torrent of big data, the clinical utility has disappointed; few cancers possess truly ‘actionable mutations’ ” [41,120].

In contrast, the speciation theory predicts that individual quasi-clonal karyotypes cause and maintain cancers, and thus explains the modest progress made in the name of the mutation theory. 

In addition to our current study, there is a large background of earlier cytogenetic studies, which have provided: (1) clinically confirmed valid prognoses of cancer risks, based on preneoplastic karyotypes; and (2) clinically confirmed valid diagnoses of malignancy based on individual clonal karyotypes. For example, Papanicolaou et al. first identified preneoplastic and neoplastic karyotypes in 1952 based on individual “DNA contents” of cervical tissues, diagnosed in “Pap smears” [62]. Subsequently other cytogeneticists established numerous consistent correlations between: (i) the degrees of preneoplastic aneuploidy and the risk for cancers; and (ii) the degrees of neoplastic aneuploidy and the degrees of malignancy of cancers [89,121,122,123,124,125,126,127,128,129,130,131,132,133,134,135,136,137,138,139,140]. 

Unexpectedly, a recent study by Lee et al. lends independent support to our conclusion about the clinical relevance of cancer cytogenetics. Lee et al. proposed that “CIN(+) predicted worse progression-free or disease-free survival relative to patients with CIN(-) disease” [141].

In view of this, we propose that preneoplastic, non-clonal karyotypes should provide absolute indicators for the risk of cancer, and that individual clonal karyotypes of cancers should provide reliable, stable and verifiable prognoses for individual cancers—independent of the “bewildering” numbers of mutations currently found in preneoplastic tissues and cancers [29].

## Figures and Tables

**Table 1:** Chromosome copy numbers of rat mammary tissue 3 months after injection with NMU.

Karyotypes	1	2	3	4	5	6	7	8	9	10	11	12	13	14	15	16	17	18
No of Chrs.	42	42	42	42	42	42	42	42	42	42	40	35	39	40	37	41	41	46
Chromosomes	Chromosome Copy Number																	
1	2	2	2	2	2	2	2	2	2	2	2	1	2	2	2	2	2	2
2	2	2	2	2	2	2	2	2	2	2	2	2	2	2	2	2	2	3
3	2	2	2	2	2	2	2	2	2	2	2	1	2	2	2	2	2	2
4	2	2	2	2	2	2	2	2	2	2	2	1	2	2	2	2	2	2
5	2	2	2	2	2	2	2	2	2	3	2	1	2	2	2	2	2	2
6	2	2	2	2	2	2	2	2	2	2	1	1	1	2	2	2	2	2
7	2	2	2	2	2	2	2	2	2	2	2	2	2	2	1	2	2	2
8	2	2	2	2	2	2	2	2	2	3	2	2	2	2	1	2	2	3
9	2	2	2	2	2	2	2	2	2	2	2	2	2	2	1	2	2	3
10	2	2	2	2	2	2	2	2	2	3	2	2	2	2	2	2	2	2
11	2	2	2	2	2	2	2	2	2	2	2	2	2	2	2	2	1	2
12	2	2	2	2	2	2	2	2	2	2	2	1	1	1	2	2	2	2
13	2	2	2	2	2	2	2	2	2	2	2	2	1	1	1	2	2	3
14	2	2	2	2	2	2	2	2	2	2	2	2	2	2	2	2	2	2
15	2	2	2	2	2	2	2	2	2	1	2	2	2	2	2	2	2	2
16	2	2	2	2	2	2	2	2	2	2	2	2	2	2	2	2	2	1
17	2	2	2	2	2	2	2	2	2	2	2	2	2	2	1	1	2	3
18	2	2	2	2	2	2	2	2	2	1	2	1	2	2	2	2	2	2
19	2	2	2	2	2	2	2	2	2	2	2	2	2	2	2	2	2	2
20	2	2	2	2	2	2	2	2	2	1	1	2	2	2	2	2	2	2
X	2	2	2	2	2	2	2	2	2	2	2	2	2	2	2	2	2	2
Y	0	0	0	0	0	0	0	0	0	0	0	0	0	0	0	0	0	0

**Table:** 

(a)																				
Karyotypes 1 Month Post-SV40	1	2	3	4	5	6	7	8	9	10	11	12	13	14	15	16	17	18	19	20
No. of Chrs.	46	46	46	46	46	46	46	46	45	45	47	49	45	45	47	47	44	48	46	42
Chromosomes	Chromosome Copy Number																			
1	2	2	2	2	2	2	2	2	2	2	2	2	1	2	2	2	2	2	2	2
2	2	2	2	2	2	2	2	2	2	2	2	2	2	2	2	2	3	2	2	2
3	2	2	2	2	2	2	2	2	2	2	2	2	2	2	2	2	2	2	2	1
4	2	2	2	2	2	2	2	2	2	2	2	2	2	2	2	2	2	2	2	2
5	2	2	2	2	2	2	2	2	2	2	2	2	2	2	3	3	2	2	3	2
6	2	2	2	2	2	2	2	2	2	2	2	2	2	2	2	2	2	2	2	1
7	2	2	2	2	2	2	2	2	2	2	2	2	2	1	2	2	2	2	2	2
8	2	2	2	2	2	2	2	2	2	2	2	2	2	2	2	2	2	2	2	1
9	2	2	2	2	2	2	2	2	2	2	2	2	2	2	2	2	2	3	2	2
10	2	2	2	2	2	2	2	2	2	2	2	2	2	2	2	2	2	2	2	2
11	2	2	2	2	2	2	2	2	2	2	2	2	2	2	2	2	2	2	1	2
12	2	2	2	2	2	2	2	2	2	2	2	2	2	2	2	2	2	2	2	2
13	2	2	2	2	2	2	2	2	2	2	2	2	2	2	2	2	2	2	3	2
14	2	2	2	2	2	2	2	2	2	2	2	2	2	2	2	2	2	2	2	2
15	2	2	2	2	2	2	2	2	2	2	3	2	2	2	2	2	2	2	2	2
16	2	2	2	2	2	2	2	2	2	2	2	2	2	2	2	2	1	2	2	2
17	2	2	2	2	2	2	2	2	2	2	2	3	2	2	2	2	1	2	2	2
18	2	2	2	2	2	2	2	2	2	2	2	2	2	2	2	2	2	2	1	2
19	2	2	2	2	2	2	2	2	2	2	2	3	2	2	2	2	2	2	2	2
20	2	2	2	2	2	2	2	2	2	2	2	2	2	2	2	2	1	3	2	2
21	2	2	2	2	2	2	2	2	1	2	2	3	2	1	2	2	1	2	2	2
22	2	2	2	2	2	2	2	2	2	2	2	2	2	2	2	2	2	2	2	2
X	2	2	2	2	2	2	2	2	2	1	2	2	2	2	2	2	2	2	2	1
Y	0	0	0	0	0	0	0	0	0	0	0	0	0	0	0	0	0	0	0	0
der(7;21)?	0	0	0	0	0	0	0	0	0	0	0	0	0	1	0	0	0	0	0	0
der(7)	0	0	0	0	0	0	0	1	0	0	0	0	0	0	0	0	0	0	0	0
i(21q)	0	0	0	0	0	0	0	0	0	0	0	0	0	0	0	0	1	0	0	0

**Table:** 

(b)																				
Karyotypes 2 Months Post-SV40	1	2	3	4	5	6	7	8	9	10	11	12	13	14	15	16	17	18	19	20
No. of Chrs.	46	45	46	46	43	46	46	47	47	46	47	47	47	47	48	49	60	75	75	83
Chromosomes	Chromosome Copy Number																			
1	2	2	2	2	2	2	2	2	2	1	1	2	2	2	2	2	2	3	4	4
2	2	1	2	2	2	2	2	2	2	2	2	2	2	2	2	2	3	1	4	3
3	2	2	2	2	2	2	2	2	2	2	2	3	2	2	2	2	2	3	4	3
4	2	2	2	2	2	2	2	2	2	2	2	2	2	2	3	2	4	2	3	4
5	2	2	2	2	2	3	2	3	2	2	1	2	2	3	2	3	1	5	3	4
6	2	1	2	2	2	2	2	2	3	2	2	2	2	2	3	2	2	3	4	2
7	2	2	2	2	2	2	2	2	2	2	2	2	2	2	2	2	2	4	2	3
8	2	2	2	2	2	2	2	2	2	2	2	2	2	2	2	2	3	3	2	3
9	2	2	2	2	2	2	2	2	2	1	2	2	2	2	2	2	3	3	2	3
10	2	2	1	2	2	2	2	2	2	2	3	2	2	2	2	2	2	4	4	4
11	2	2	2	2	2	2	2	2	2	2	2	2	2	2	2	2	2	3	3	3
12	2	2	2	2	2	2	2	2	2	2	2	2	2	2	2	2	3	4	3	4
13	2	2	2	2	2	2	2	2	2	2	2	2	2	2	2	2	2	4	3	3
14	2	2	2	1	1	1	2	2	2	2	2	2	2	2	2	2	2	4	4	4
15	2	2	2	2	1	2	1	2	2	2	1	2	2	2	2	2	4	4	3	4
16	2	2	2	2	1	2	2	2	2	2	2	2	2	2	2	2	4	2	3	2
17	2	2	2	2	2	1	2	2	2	2	2	2	2	2	2	2	1	4	4	3
18	2	2	2	2	2	2	2	2	2	2	2	2	2	1	3	2	2	3	2	2
19	2	2	2	2	2	2	2	2	2	1	1	2	2	2	1	3	2	3	3	4
20	2	2	1	2	2	2	2	2	2	2	2	2	2	2	2	3	4	4	4	4
21	2	2	2	2	2	2	2	2	2	2	2	2	2	2	2	2	1	2	3	3
22	2	2	2	2	2	2	2	2	2	2	2	2	2	2	2	2	4	2	3	2
X	2	2	2	2	2	2	2	2	2	2	2	2	2	2	2	2	1	3	3	4
Y	0	0	0	0	0	0	0	0	0	0	0	0	0	0	0	0	0	0	0	0
der(1q)	0	0	0	0	0	0	0	0	0	1	1	0	0	0	0	0	0	0	0	0
der(18) long	0	0	0	0	0	0	0	0	0	0	0	0	0	1	0	0	0	0	1	0
der(9;6;9;6)	0	0	0	0	0	0	0	0	0	0	0	0	0	0	0	0	0	0	0	1
der(17;18)	0	0	0	0	0	0	0	0	0	0	0	0	0	0	0	0	0	0	0	1
der(22;7)	0	0	0	0	0	0	0	0	0	0	0	0	0	0	0	0	0	0	0	1
der(16;8;3)	0	0	0	0	0	0	0	0	0	0	0	0	0	0	0	0	0	0	0	1
der(8;3)	0	0	0	0	0	0	0	0	0	0	0	0	0	0	0	0	0	0	0	1
der(22;16)	0	0	0	0	0	0	0	0	0	0	0	0	0	0	0	0	0	0	0	1
min(3?)	0	0	0	0	0	0	0	0	0	0	0	0	0	0	0	0	0	0	0	1
der(8)	0	0	0	0	0	0	0	0	0	0	0	0	0	0	0	0	0	0	0	1
der(1;17)	0	0	0	0	0	0	0	0	0	0	0	0	0	0	0	0	1	0	0	0
der(17;12)	0	0	0	0	0	0	0	0	0	0	0	0	0	0	0	0	1	0	0	0
der(22) small	0	0	0	0	0	0	0	0	0	0	0	0	0	0	0	0	1	0	0	0
min(19)	0	0	0	0	0	0	0	0	0	0	0	0	0	0	0	0	1	0	0	0
der(19q)	0	0	0	0	0	0	0	0	0	1	0	0	0	0	0	0	0	0	0	0
der(9;13?;9)	0	0	0	0	0	0	0	0	0	1	0	0	0	0	0	0	0	0	0	0
der(16q)	0	0	0	0	0	0	0	0	0	0	0	0	0	0	0	0	0	2	0	0
der(19;14)	0	0	0	0	0	0	0	0	0	0	1	0	0	0	0	0	0	0	0	0
der(1)	0	0	0	0	0	0	0	0	0	0	1	0	0	0	0	0	0	0	0	0
dic(5;15)	0	0	0	0	0	0	0	0	0	0	1	0	0	0	0	0	0	0	0	0
i(14q)	0	0	0	1	0	0	0	0	0	0	0	0	0	0	0	0	0	0	0	0
dic(2;6)	0	1	0	0	0	0	0	0	0	0	0	0	0	0	0	0	0	0	0	0
der(13) small	0	0	0	0	0	0	0	0	0	0	0	0	1	0	0	0	0	0	0	0
der(15;3)	0	0	0	0	0	0	1	0	0	0	0	0	0	0	0	0	0	0	0	0
der(12;9)	0	0	0	0	0	0	0	0	0	0	0	0	0	0	0	0	0	0	1	0
der(10q)	0	0	1	0	0	0	0	0	0	0	0	0	0	0	0	0	0	0	0	0
der(20) long	0	0	1	0	0	0	0	0	0	0	0	0	0	0	0	0	0	0	0	0
der(17)	0	0	0	0	0	1	0	0	0	0	0	0	0	0	0	0	0	0	0	0

**Table:** 

(c)																				
Karyotypes 6 Months Post-SV40	1	2	3	4	5	6	7	8	9	10	11	12	13	14	15	16	17	18	19	20
No. of Chrs.	44	40	47	43	44	43	45	41	44	45	44	45	47	43	42	51	65	83	73	84
Chromosomes	Chromosome Copy Number																			
1	2	2	2	2	2	2	2	2	2	2	2	2	1	2	2	2	3	4	4	3
2	2	2	2	2	1	2	2	2	2	2	2	2	2	1	2	3	4	4	2	3
3	2	1	2	2	1	2	2	1	2	2	1	2	1	1	2	2	2	2	4	4
4	2	2	2	2	1	2	2	2	2	1	2	2	3	2	2	2	4	4	2	3
5	3	3	3	3	3	3	2	1	2	2	1	2	2	1	3	2	2	2	3	4
6	2	0	2	1	2	2	2	2	2	2	1	2	1	2	2	2	2	2	3	5
7	2	2	2	1	2	1	1	1	2	2	2	2	1	2	2	1	1	4	0	2
8	2	2	2	2	2	2	2	2	2	1	1	2	2	1	2	1	3	2	4	2
9	2	1	2	2	2	2	2	1	2	1	2	1	1	2	2	2	2	4	3	5
10	2	1	2	2	1	2	1	2	2	2	2	1	1	2	2	2	3	4	4	4
11	2	2	2	1	1	2	2	2	2	2	2	2	2	2	2	1	3	2	3	4
12	2	2	1	1	1	1	2	0	2	1	2	1	2	1	1	2	3	4	4	2
13	2	2	2	2	2	1	1	1	1	2	2	2	2	1	1	1	3	4	1	3
14	2	2	2	2	2	2	2	2	2	2	2	3	3	2	2	2	2	4	2	2
15	1	1	2	1	2	1	1	1	1	1	2	1	2	1	2	2	1	2	2	2
16	2	2	2	2	2	1	1	2	2	2	2	2	2	1	2	2	3	4	2	4
17	2	1	2	1	2	2	2	0	1	1	2	2	2	2	2	2	1	4	2	4
18	2	2	2	2	2	2	1	2	2	2	1	1	1	2	1	2	4	4	2	3
19	1	2	2	1	2	1	2	2	2	2	2	2	1	2	2	1	4	2	2	3
20	2	2	2	1	2	2	2	1	2	2	2	2	1	2	2	1	2	2	2	3
21	1	1	2	2	1	2	1	1	1	1	1	1	1	1	0	0	2	2	2	3
22	0	0	2	1	1	1	1	0	2	1	1	1	2	1	1	2	0	2	2	3
X	2	2	2	2	2	2	2	2	2	2	2	2	2	2	2	2	3	4	4	3
Y	0	0	0	0	0	0	0	0	0	0	0	0	0	0	0	0	0	0	0	0
der(15;22)	1	1	0	1	0	1	0	0	0	0	0	1	0	0	0	0	0	2	0	0
der(19;21)	1	0	0	0	0	0	0	0	0	0	0	0	0	0	0	1	0	0	0	0
der(6;21)	0	1	0	0	0	0	0	0	0	0	0	0	0	0	0	0	0	0	0	0
der(22;6)	0	1	0	0	0	0	0	0	0	0	0	0	0	0	0	0	0	0	0	0
der(12;9)	0	0	1	0	0	0	0	0	0	0	0	0	0	0	0	0	0	0	0	0
der(6;8)	0	0	0	1	0	0	0	0	0	0	0	0	0	0	0	0	0	0	0	0
der(11;20)	0	0	0	1	0	0	0	0	0	0	0	0	0	0	0	0	0	0	0	0
dic(12;19)	0	0	0	1	0	0	0	0	0	0	0	0	0	0	0	0	0	0	0	0
dic(7;17)	0	0	0	1	0	0	0	0	0	0	0	0	0	0	0	0	0	0	0	0
dic(4;10)	0	0	0	0	1	0	0	0	0	0	0	0	0	0	0	0	0	0	0	0
der(3;21)	0	0	0	0	1	0	0	0	0	0	1	0	0	0	0	0	0	0	0	0
der(2;22)	0	0	0	0	1	0	0	0	0	0	0	0	0	0	0	0	0	0	0	0
der(11;12)	0	0	0	0	1	0	0	0	0	0	0	0	0	0	0	0	0	0	0	0
del(2q)	0	0	0	0	1	0	0	0	0	0	0	0	0	0	0	0	0	0	0	0
der(7;12)	0	0	0	0	0	1	0	0	0	0	0	0	0	0	0	0	0	0	0	0
dic(16;19)	0	0	0	0	0	1	0	0	0	0	0	0	0	0	0	0	0	0	0	0
der(7;15)	0	0	0	0	0	0	1	0	0	0	0	0	0	0	0	0	0	0	0	0
der(7;22)	0	0	0	0	0	0	1	0	0	0	0	0	0	0	0	0	0	0	0	0
der(12;20;21)	0	0	0	0	0	0	1	0	0	0	0	0	0	0	0	0	0	0	0	0
dic(12;16)	0	0	0	0	0	0	1	0	0	0	0	0	0	0	0	0	0	0	0	0
der(5;9)	0	0	0	0	0	0	1	0	0	0	0	0	0	0	0	0	0	0	0	0
der(18;20)	0	0	0	0	0	0	1	0	0	0	0	0	0	0	0	0	0	0	0	0
der(16)	0	0	0	0	0	0	1	0	0	0	0	0	0	0	0	0	0	0	0	0
dic(5;9)	0	0	0	0	0	0	0	1	0	0	0	0	0	0	0	0	0	0	0	0
dic(5;22)	0	0	0	0	0	0	0	1	0	0	0	0	0	0	0	0	0	0	0	0
der(15;7)	0	0	0	0	0	0	0	1	0	0	0	0	0	0	0	0	0	0	0	0
dic(16;20)	0	0	0	0	0	0	0	1	0	0	0	0	0	0	0	0	0	0	0	0
dic(12;22;17)	0	0	0	0	0	0	0	1	0	0	0	0	0	0	0	0	0	0	0	0
der(12;7)	0	0	0	0	0	0	0	1	0	0	0	0	0	0	0	0	0	0	0	0
der(10;22)	0	0	0	0	0	0	0	1	0	0	0	0	0	0	0	0	0	0	0	0
der(10;21)	0	0	0	0	0	0	0	1	0	0	0	0	0	0	0	0	0	0	0	0
der(3)	0	0	0	0	0	0	0	1	0	0	0	0	0	0	0	0	0	0	0	0
der(5;15)	0	0	0	0	0	0	0	0	1	1	0	0	0	0	0	0	0	0	0	0
der(17;21)	0	0	0	0	0	0	0	0	1	0	0	0	0	0	0	0	0	0	0	0
der(8;21)	0	0	0	0	0	0	0	0	0	1	0	0	0	0	0	0	0	0	0	0
der(4;17)	0	0	0	0	0	0	0	0	0	1	0	0	0	0	0	0	0	0	0	0
del(4p)	0	0	0	0	0	0	0	0	0	1	0	0	0	0	0	0	0	0	1	0
der(5;12)	0	0	0	0	0	0	0	0	0	1	0	0	0	0	0	0	0	0	0	0
der(12)	0	0	0	0	0	0	0	0	0	1	0	0	0	0	0	0	0	0	0	0
del(9q)	0	0	0	0	0	0	0	0	0	1	0	0	0	0	0	0	0	0	0	0
dic(5;6)	0	0	0	0	0	0	0	0	0	0	1	0	0	0	0	0	0	0	0	0
dic(5;22)long	0	0	0	0	0	0	0	0	0	0	1	0	0	0	0	0	0	0	0	0
i(18)	0	0	0	0	0	0	0	0	0	0	1	0	0	0	0	0	0	0	0	0
der(3;21)	0	0	0	0	0	0	0	0	0	0	1	0	0	0	0	0	0	0	0	0
der(9;11)	0	0	0	0	0	0	0	0	0	0	0	1	0	0	0	0	0	0	0	0
der(5;21)	0	0	0	0	0	0	0	0	0	0	0	1	0	0	0	0	0	0	0	0
der(12;10)	0	0	0	0	0	0	0	0	0	0	0	1	0	0	0	0	0	0	0	0
der(10;12)	0	0	0	0	0	0	0	0	0	0	0	1	0	0	0	0	0	0	0	0
der(7;18)	0	0	0	0	0	0	0	0	0	0	0	0	1	0	0	0	0	0	0	0
der(4;22)	0	0	0	0	0	0	0	0	0	0	0	0	1	0	0	0	0	0	0	0
der(19;11)	0	0	0	0	0	0	0	0	0	0	0	0	1	0	0	0	0	0	0	0
der(5)short	0	0	0	0	0	0	0	0	0	0	0	0	1	0	0	0	0	0	0	0
der(9;15)	0	0	0	0	0	0	0	0	0	0	0	0	1	0	0	0	0	0	0	0
dic(20;6;21)	0	0	0	0	0	0	0	0	0	0	0	0	1	0	0	0	0	0	0	0
del(6q)	0	0	0	0	0	0	0	0	0	0	0	0	1	0	0	0	0	0	0	0
der(1)	0	0	0	0	0	0	0	0	0	0	0	0	1	0	0	0	0	0	0	0
der(21;17)	0	0	0	0	0	0	0	0	0	0	0	0	1	0	0	0	0	0	0	0
der(2;5)	0	0	0	0	0	0	0	0	0	0	0	0	0	1	0	0	0	0	0	0
der(3;15)	0	0	0	0	0	0	0	0	0	0	0	0	0	1	0	0	0	0	0	0
der(16;21)	0	0	0	0	0	0	0	0	0	0	0	0	0	1	0	0	0	0	1	0
(der(8;11)	0	0	0	0	0	0	0	0	0	0	0	0	0	1	0	0	0	0	0	0
der(8;12)	0	0	0	0	0	0	0	0	0	0	0	0	0	1	0	0	0	0	0	0
der(X;22)	0	0	0	0	0	0	0	0	0	0	0	0	0	1	0	0	0	0	0	0
del(5q)	0	0	0	0	0	0	0	0	0	0	0	0	0	1	0	0	0	0	0	0
der(21;22)	0	0	0	0	0	0	0	0	0	0	0	0	0	0	1	0	0	0	0	0
der(5;6)	0	0	0	0	0	0	0	0	0	0	0	0	0	0	0	1	0	0	0	0
der(8;13)	0	0	0	0	0	0	0	0	0	0	0	0	0	0	0	1	0	0	0	0
der(6;8;14)	0	0	0	0	0	0	0	0	0	0	0	0	0	0	0	1	0	0	0	0
der(17;6)	0	0	0	0	0	0	0	0	0	0	0	0	0	0	0	1	0	0	0	0
min(16)	0	0	0	0	0	0	0	0	0	0	0	0	0	0	0	1	0	0	0	0
der(11;14)	0	0	0	0	0	0	0	0	0	0	0	0	0	0	0	1	0	0	0	0
der(6;8)	0	0	0	0	0	0	0	0	0	0	0	0	0	0	0	1	0	0	0	0
der(7;5;21)	0	0	0	0	0	0	0	0	0	0	0	0	0	0	0	1	0	0	0	0
der(2;5)short	0	0	0	0	0	0	0	0	0	0	0	0	0	0	0	1	0	0	0	0
der(20;6)	0	0	0	0	0	0	0	0	0	0	0	0	0	0	0	1	0	0	0	0
mar(12)	0	0	0	0	0	0	0	0	0	0	0	0	0	0	0	1	0	0	0	0
der(9;10)	0	0	0	0	0	0	0	0	0	0	0	0	0	0	0	0	1	0	0	0
der(17;22)	0	0	0	0	0	0	0	0	0	0	0	0	0	0	0	0	1	0	0	0
der(21;7)	0	0	0	0	0	0	0	0	0	0	0	0	0	0	0	0	1	0	0	0
der(3;22)	0	0	0	0	0	0	0	0	0	0	0	0	0	0	0	0	1	0	0	0
dic(X;6)	0	0	0	0	0	0	0	0	0	0	0	0	0	0	0	0	1	0	0	0
der(5;20;21)	0	0	0	0	0	0	0	0	0	0	0	0	0	0	0	0	1	0	0	0
del(5q)short	0	0	0	0	0	0	0	0	0	0	0	0	0	0	0	0	1	0	0	0
der(17)long	0	0	0	0	0	0	0	0	0	0	0	0	0	0	0	0	1	0	0	0
der(3;5)	0	0	0	0	0	0	0	0	0	0	0	0	0	0	0	0	0	2	0	0
dic(5;6)short	0	0	0	0	0	0	0	0	0	0	0	0	0	0	0	0	0	2	0	0
dic(8;11)	0	0	0	0	0	0	0	0	0	0	0	0	0	0	0	0	0	2	0	0
der(20;21)	0	0	0	0	0	0	0	0	0	0	0	0	0	0	0	0	0	2	0	0
der(19;5;19)	0	0	0	0	0	0	0	0	0	0	0	0	0	0	0	0	0	1	0	0
der(18;2)	0	0	0	0	0	0	0	0	0	0	0	0	0	0	0	0	0	0	1	0
der(2;3)	0	0	0	0	0	0	0	0	0	0	0	0	0	0	0	0	0	0	1	0
der(12;2;19)	0	0	0	0	0	0	0	0	0	0	0	0	0	0	0	0	0	0	1	0
der(4;11;4)	0	0	0	0	0	0	0	0	0	0	0	0	0	0	0	0	0	0	1	0
der(5;17)	0	0	0	0	0	0	0	0	0	0	0	0	0	0	0	0	0	0	1	0
der(7;21)	0	0	0	0	0	0	0	0	0	0	0	0	0	0	0	0	0	0	1	0
der(10;19)	0	0	0	0	0	0	0	0	0	0	0	0	0	0	0	0	0	0	1	0
der(14;22)	0	0	0	0	0	0	0	0	0	0	0	0	0	0	0	0	0	0	1	0
der(17;14)	0	0	0	0	0	0	0	0	0	0	0	0	0	0	0	0	0	0	1	0
del(7q)	0	0	0	0	0	0	0	0	0	0	0	0	0	0	0	0	0	0	1	0
der(14;15)	0	0	0	0	0	0	0	0	0	0	0	0	0	0	0	0	0	0	1	0
der(16)	0	0	0	0	0	0	0	0	0	0	0	0	0	0	0	0	0	0	1	0
der(1;18)	0	0	0	0	0	0	0	0	0	0	0	0	0	0	0	0	0	0	0	1
der(1;16)	0	0	0	0	0	0	0	0	0	0	0	0	0	0	0	0	0	0	0	1
der(6;14)	0	0	0	0	0	0	0	0	0	0	0	0	0	0	0	0	0	0	0	1
der(6;7)	0	0	0	0	0	0	0	0	0	0	0	0	0	0	0	0	0	0	0	1
der(5;7)	0	0	0	0	0	0	0	0	0	0	0	0	0	0	0	0	0	0	0	1
der(8;13)short	0	0	0	0	0	0	0	0	0	0	0	0	0	0	0	0	0	0	0	1
der(5;9)short	0	0	0	0	0	0	0	0	0	0	0	0	0	0	0	0	0	0	0	1
der(16;18)	0	0	0	0	0	0	0	0	0	0	0	0	0	0	0	0	0	0	0	1
dic(5;2;17;9)	0	0	0	0	0	0	0	0	0	0	0	0	0	0	0	0	0	0	0	1
der(X;21)	0	0	0	0	0	0	0	0	0	0	0	0	0	0	0	0	0	0	0	1

**Table:** 

(a)																														
F10 Karyotypes	1	2	3	4	5	6	7	8	9	10	11	12	13	14	15	16	17	18	19	20	21	22	23	24	25	26	27	28	29	30
No. of Chrs.	41	41	41	41	39	42	42	41	41	41	40	42	41	42	40	42	41	41	41	42	43	41	41	41	39	41	42	81	76	84
Chromosomes	Chromosome Copy Number																													
1	2	1	2	2	2	2	2	2	2	2	2	2	2	2	2	2	2	2	2	2	2	2	2	2	1	2	2	4	3	4
2	2	2	2	2	2	2	2	2	2	2	2	2	2	2	2	2	2	2	2	2	2	2	2	2	2	2	2	3	4	4
3	1	0	1	1	0	1	1	1	1	2	1	0	1	1	1	1	2	1	1	2	1	1	1	1	1	1	1	3	2	3
4	2	2	2	2	1	2	2	2	2	2	2	2	2	2	2	2	2	2	2	2	2	2	2	2	2	2	2	4	3	3
5	1	1	2	1	1	2	2	2	2	2	1	2	1	2	1	1	1	1	1	1	1	2	1	2	1	2	1	2	2	4
6	2	2	2	2	1	2	2	2	2	2	2	2	2	2	2	2	1	2	1	2	1	2	2	2	2	2	2	4	4	4
7	2	2	2	2	1	2	2	2	2	2	2	2	2	2	2	2	2	2	2	2	2	2	2	2	2	1	2	4	4	3
8	2	2	2	2	2	2	2	2	2	2	2	2	2	2	2	2	2	2	2	2	2	2	2	2	2	2	2	4	4	4
9	2	2	2	2	2	2	2	2	2	2	2	2	2	2	2	2	2	2	2	2	2	2	2	2	2	2	2	4	4	4
10	2	2	2	2	2	2	2	2	2	2	2	2	2	1	2	2	2	2	2	2	2	2	2	2	2	2	2	4	3	4
11	2	1	1	1	1	1	1	1	1	1	1	2	1	2	1	2	2	1	1	1	1	1	1	1	1	1	1	1	2	2
12	1	1	1	1	1	2	1	1	1	1	1	2	1	2	0	0	1	1	1	2	2	1	1	1	0	0	1	4	2	4
13	2	2	2	2	2	2	2	2	2	2	2	2	2	2	2	1	2	2	2	2	2	2	2	2	2	2	2	4	3	4
14	2	2	2	2	2	2	2	2	2	2	2	2	1	2	2	2	2	2	2	2	2	2	2	2	2	2	2	3	4	4
15	2	2	2	2	2	2	2	2	2	2	2	2	2	2	2	2	2	2	2	2	2	2	2	2	2	2	2	4	3	4
16	2	2	2	2	2	2	2	2	2	2	2	2	2	2	2	2	2	2	2	2	2	2	2	2	2	2	2	4	4	4
17	2	2	2	2	2	2	2	2	2	2	2	2	2	2	2	2	1	2	2	2	2	2	2	2	2	2	2	4	3	4
18	2	1	2	2	2	2	3	2	2	2	2	2	2	2	2	2	2	2	2	2	2	2	2	2	2	2	2	4	4	4
19	2	2	2	2	2	2	2	2	1	2	2	2	2	2	2	2	2	2	2	2	2	2	2	2	2	2	2	4	4	3
20	2	2	2	2	2	2	2	2	2	2	2	2	2	2	2	2	2	2	2	2	2	2	2	2	2	2	2	4	4	4
X	2	2	2	2	2	2	2	2	2	2	2	2	2	2	2	2	2	2	2	2	2	2	2	2	2	2	2	4	4	3
Y	0	0	0	0	0	0	0	0	0	0	0	0	0	0	0	0	0	0	0	0	0	0	0	0	0	0	0	0	0	0
der(3;12)	1	1	1	0	1	0	0	0	0	0	0	0	1	0	1	1	0	1	0	0	0	0	1	0	1	1	0	0	2	0
i(5q)	0	0	0	0	0	1	0	0	0	0	1	1	1	0	0	1	1	0	0	0	0	0	0	0	0	0	0	2	0	0
der(5;11)	0	0	1	0	1	0	0	0	0	1	0	0	0	1	1	0	0	1	0	0	0	0	1	0	1	0	0	0	2	0
der(3;11)	0	0	0	1	0	1	1	0	0	0	1	0	0	0	0	0	0	0	0	0	0	0	0	0	0	0	0	2	0	1
der(5;12)	0	0	0	1	0	0	1	0	0	0	0	0	0	0	0	1	1	0	0	0	0	0	0	0	0	0	0	0	0	0
der(3;5)	0	0	0	0	0	0	0	1	0	0	0	0	1	1	0	1	0	0	0	0	0	0	0	1	0	0	0	0	0	0
der(5;5)	1	0	0	0	0	0	0	0	0	0	0	0	0	0	0	0	0	1	0	1	0	0	1	0	1	1	0	0	0	0
der(5;9)	0	1	0	0	0	0	0	0	0	0	0	0	0	0	0	0	0	0	0	0	0	0	0	0	0	0	0	0	0	0
der(11;12)	0	0	0	0	0	0	0	1	0	0	0	0	0	0	0	0	0	0	0	0	0	0	0	1	0	0	0	0	0	0
der(5;7)	0	0	0	0	0	0	0	0	0	0	0	0	0	0	0	0	0	0	0	0	0	0	0	0	0	0	0	0	0	1
der(5)	0	0	0	0	0	0	0	0	0	0	0	0	0	0	0	1	0	0	0	1	0	0	0	0	0	0	0	0	0	0
der(3;1;19)	0	1	0	0	0	0	0	0	0	0	0	0	0	0	0	0	0	0	0	0	0	0	0	0	0	0	0	0	0	0
dic(1;5;5)	0	1	0	0	0	0	0	0	0	0	0	0	0	0	0	0	0	0	0	0	0	0	0	0	0	0	0	0	0	0
der(1;5)	0	1	0	0	0	0	0	0	0	0	0	0	0	0	0	0	0	0	0	0	0	0	0	0	0	0	0	0	0	0
der(1;11)	0	1	0	0	0	0	0	0	0	0	0	0	0	0	0	0	0	0	0	0	0	0	0	0	0	0	0	0	0	0
dic(4;6)	0	0	0	0	1	0	0	0	0	0	0	0	0	0	0	0	0	0	0	0	0	0	0	0	0	0	0	0	0	0
dic(3;5;5)	0	0	0	0	1	0	0	0	0	0	0	0	0	0	0	0	0	0	0	0	0	0	0	0	0	0	0	0	0	0
der(7)	0	0	0	0	1	0	0	0	0	0	0	0	0	0	0	0	0	0	0	0	0	0	0	0	0	0	0	0	0	0
der(i(5q);11)	0	0	0	0	0	0	0	0	1	0	0	0	0	0	0	0	0	0	0	0	0	0	0	0	0	0	0	0	0	0
der(1;3)	0	0	0	0	0	0	0	0	1	0	0	0	0	0	0	0	0	0	0	0	0	0	0	0	0	0	0	0	0	0
der(12;19)	0	0	0	0	0	0	0	0	1	0	0	0	0	0	0	0	0	0	0	0	0	0	0	0	0	0	0	0	0	0
i(3q)	0	0	0	0	0	0	0	0	0	0	0	1	0	0	0	0	0	0	0	0	0	0	0	0	0	0	0	0	0	0
der(11;1)	0	0	0	0	0	0	0	0	0	0	0	0	1	0	0	0	0	0	0	0	0	0	0	0	0	0	0	0	0	0
dic(5;12)	0	0	0	0	0	0	0	0	0	0	0	0	0	0	1	0	0	0	1	0	0	1	0	0	0	0	1	0	0	0
dic(6;17)	0	0	0	0	0	0	0	0	0	0	0	0	0	0	0	0	1	0	0	0	0	0	0	0	0	0	0	0	0	0
dic(2;5)	0	0	0	0	0	0	0	0	0	0	0	0	0	0	0	0	0	0	0	0	0	0	0	0	0	0	0	1	0	0
dic(5;13)	0	0	0	0	0	0	0	0	0	0	0	0	0	0	0	0	0	0	0	0	0	0	0	0	0	0	0	0	1	0
der(3;10)	0	0	0	0	0	0	0	0	0	0	0	0	0	0	0	0	0	0	0	0	0	0	0	0	0	0	0	0	1	0
tricentric(5;5;5)	0	0	0	1	0	0	0	0	0	0	0	0	0	0	0	0	0	0	0	0	0	0	0	0	0	0	0	0	0	0
der(X;4)	0	0	0	0	0	0	0	0	0	0	0	0	0	0	0	0	0	0	0	0	0	0	0	0	0	0	0	0	0	1
der(5;19)	0	0	0	0	0	0	0	0	0	0	0	0	0	0	0	0	0	0	0	0	0	0	0	0	0	0	0	0	0	1
der(19;5)	0	0	0	0	0	0	0	0	0	0	0	0	0	0	0	0	0	0	0	0	0	0	0	0	0	0	0	0	0	1
i(4q)	0	0	0	0	0	0	0	0	0	0	0	0	0	0	0	0	0	0	0	0	0	0	0	0	0	0	0	0	0	1
der(4)	0	0	0	0	0	0	0	0	0	0	0	0	0	0	0	0	0	0	0	0	0	0	0	0	0	0	0	0	0	1
der(5,6)	0	0	0	0	0	0	0	0	0	0	0	0	0	0	0	0	0	0	1	0	1	0	0	0	0	0	0	0	0	0
der(3,11)	0	0	0	0	0	0	0	0	0	0	0	0	0	0	0	0	0	0	1	0	1	1	0	0	0	0	1	0	0	0
der(1,5,6)	0	0	0	0	0	0	0	0	0	0	0	0	0	0	0	0	0	0	1	0	0	0	0	0	0	0	0	0	0	0
der(6,9,6)	0	0	0	0	0	0	0	0	0	0	0	0	0	0	0	0	0	0	0	0	1	0	0	0	0	0	0	0	0	0
der(5,6,5,20?)	0	0	0	0	0	0	0	0	0	0	0	0	0	0	0	0	0	0	0	0	1	0	0	0	0	0	0	0	0	0
der(6,5,20)	0	0	0	0	0	0	0	0	0	0	0	0	0	0	0	0	0	0	0	0	1	0	0	0	0	0	0	0	0	0
der(4,11)	0	0	0	0	0	0	0	0	0	0	0	0	0	0	0	0	0	0	0	0	0	0	0	0	0	1	0	0	0	0
der(7,12)	0	0	0	0	0	0	0	0	0	0	0	0	0	0	0	0	0	0	0	0	0	0	0	0	0	1	0	0	0	0
dic(2;6;5;6;5;6;5)	0	0	0	0	0	0	0	0	0	0	0	0	0	0	0	0	0	0	0	0	0	0	0	0	0	0	1	0	0	0
der(2;11?,5)	0	0	0	0	0	0	0	0	0	0	0	0	0	0	0	0	0	0	0	0	0	0	0	0	0	0	1	0	0	0

**Table:** 

(b)																
C1 Karyotype	1	2	3	4	5	6	7	8	9	10	11	12	13	14	15	16
No. of Chrms.	41	42	42	46	42	42	45	42	42	42	42	44	40	44	46	41
Chromosomes	Chromosome Copy Number															
1	2	2	2	2	1	2	2	2	2	2	2	2	2	2	1	2
2	1	2	2	2	2	2	2	2	2	2	1	2	2	2	2	2
3	1	1	1	1	1	1	2	1	1	1	1	1	1	1	1	1
4	2	2	2	2	2	2	2	2	2	2	2	1	1	2	2	2
5	1	1	1	2	2	2	1	1	1	1	1	2	1	1	1	1
6	2	2	2	2	2	2	2	2	2	2	2	2	2	2	2	2
7	2	2	2	2	2	2	2	2	2	2	2	2	2	2	2	1
8	2	2	2	2	2	2	2	2	2	2	2	2	2	2	2	2
9	1	1	2	2	2	2	2	2	1	1	1	1	2	1	1	1
10	2	2	2	2	2	2	2	2	2	2	2	1	2	2	2	2
11	2	1	2	2	0	0	2	1	1	0	1	1	2	1	1	1
12	0	0	0	0	0	0	0	0	0	0	0	0	0	1	0	0
13	2	2	2	1	2	2	2	2	2	2	2	2	2	2	2	2
14	2	2	2	2	2	2	2	2	2	2	2	2	2	2	2	2
15	2	2	2	2	2	2	2	2	2	2	2	2	2	2	2	2
16	2	2	2	2	2	2	2	2	2	2	2	2	2	2	2	2
17	2	2	2	2	2	1	2	2	2	2	2	2	2	2	2	2
18	2	2	2	2	2	2	2	2	2	2	2	2	2	2	2	2
19	2	2	2	2	2	2	2	2	2	2	2	2	2	2	2	2
20	2	2	2	2	2	2	2	2	2	2	2	1	2	2	2	2
X	1	1	2	2	2	2	2	1	1	1	2	1	2	1	1	1
Y	0	0	0	0	0	0	0	0	0	0	0	0	0	0	0	0
i(5q)	1	1	1	1	1	1	1	1	1	1	2	0	1	1	1	0
der(5;12)	2	2	2	1	0	1	2	2	2	2	2	2	1	2	2	2
der(12;11)	0	1	0	0	1	0	1	1	1	2	1	0	0	1	0	1
der(3;12)	0	0	1	1	1	1	0	0	0	0	1	0	1	0	1	0
der(12;3;X)	0	1	0	0	0	0	0	1	1	1	0	1	0	1	0	1
der(X;3)	0	1	0	0	0	0	0	1	1	1	0	1	0	1	1	0
der(3;9)	1	0	0	0	0	0	0	0	0	0	0	0	0	0	0	0
der(12;9;X)	1	0	0	0	0	0	0	0	0	0	0	0	0	0	0	0
mar(2)	1	0	0	0	0	0	0	0	0	0	0	0	0	0	0	0
mar(9)	0	1	0	0	0	0	0	0	0	1	0	0	0	0	0	0
mar(13)	0	0	0	1	0	0	0	0	0	0	0	0	0	0	0	0
mar(11)	0	0	0	1	0	0	0	0	0	0	0	0	0	0	0	0
mar(12)	0	0	0	1	1	1	0	0	0	0	0	0	0	0	1	0
der(11,1,5)	0	0	0	0	1	0	0	0	0	0	0	0	0	0	0	0
der(4,12)	0	0	0	0	0	1	0	0	0	0	0	0	0	0	0	0
der(4,16)	0	0	0	0	0	1	0	0	0	0	0	0	0	0	0	0
der(12,9)	0	0	0	0	0	0	0	0	1	0	0	0	0	0	0	0
der(2,5)	0	0	0	0	0	0	0	0	0	0	1	0	0	0	0	0
der(9,11)	0	0	0	0	0	0	0	0	0	0	0	0	0	0	1	0
mar(7)	0	0	0	0	0	0	0	0	0	0	0	0	0	0	0	1
der(5, 7)	0	0	0	0	0	0	0	0	0	0	0	0	0	0	0	1

**Table:** 

(c)									
C2 Karyotype	1	2	3	4	5	6	7	8	9
No. of Chrs.	43	41	42	38	41	40	40	40	43
Chromosomes	Chromosomes Copy Number								
1	2	2	1	2	2	2	1	2	2
2	3	3	2	2	3	2	2	2	3
3	1	1	0	1	1	1	1	1	1
4	2	2	2	2	2	2	2	1	2
5	1	1	1	1	1	1	1	1	2
6	3	2	2	1	2	2	2	2	2
7	2	1	2	1	1	1	1	2	1
8	2	2	1	1	2	2	2	2	2
9	1	2	1	1	1	1	2	1	2
10	2	2	1	2	2	2	2	2	2
11	1	1	1	1	1	1	1	1	1
12	0	0	0	0	0	0	0	0	0
13	2	2	3	2	2	2	2	2	2
14	2	2	3	2	2	2	2	2	2
15	2	2	2	1	2	2	1	2	2
16	2	2	2	2	2	2	2	2	2
17	2	2	2	2	2	2	2	2	2
18	2	2	2	2	2	2	2	2	2
19	2	2	2	2	2	2	2	2	2
20	2	2	2	2	2	2	2	2	2
X	2	2	2	2	2	2	2	2	2
Y	0	0	0	0	0	0	0	0	0
der(5;9)long	1	1	1	1	1	1	1	1	1
der(5;7)	1	0	1	1	1	1	1	1	1
der(11;12)	1	1	1	1	1	1	1	1	1
der(3;12)	1	1	1	1	1	1	1	1	1
der(5;7)	0	1	0	1	1	1	1	0	0
der(5)	1	0	0	0	0	0	0	0	0
der(5;5)	0	0	1	0	0	0	0	0	0
dic(1;11)	0	0	1	0	0	0	0	0	0
t(3;8)	0	0	1	0	0	0	0	0	0
t(8;3)	0	0	1	0	0	0	0	0	0
der(6;8)	0	0	0	1	0	0	0	0	0
der(1)	0	0	0	0	0	0	1	0	0
der(4;5)	0	0	0	0	0	0	0	1	0
der(10;7)	0	0	0	0	0	0	0	0	1

**Table:** 

(d)																													
C6 Karyotype	1	2	3	4	5	6	7	8	9	10	11	12	13	14	15	16	17	18	19	20	21	22	23	24	25	26	27	28	29
No. of Chrs.	42	41	42	41	41	41	41	41	42	37	41	43	42	43	39	42	42	43	42	42	43	42	43	39	42	42	43	42	42
Chromosomes	Chromosome Copy Number																												
1	2	2	2	1	2	2	2	2	2	2	2	2	2	2	2	2	2	2	2	2	2	2	2	2	2	2	2	2	2
2	2	2	2	2	2	2	2	2	2	2	1	2	2	2	2	2	2	2	2	2	2	2	2	2	2	2	2	2	2
3	1	0	1	1	1	1	1	1	1	1	1	2	1	1	1	2	1	1	1	2	2	1	1	1	2	1	1	1	2
4	2	2	2	2	2	2	2	2	2	1	2	2	2	2	1	2	2	2	2	2	2	2	2	1	2	2	2	2	2
5	1	1	1	1	1	1	1	1	1	1	1	1	1	1	1	1	1	1	1	1	1	1	1	1	1	1	1	1	1
6	2	2	2	2	2	2	2	2	2	2	2	2	2	2	2	2	2	2	2	2	2	2	2	2	2	2	2	2	2
7	2	2	2	2	2	2	2	2	2	2	2	2	2	2	2	1	2	2	2	2	2	2	2	2	1	2	2	2	2
8	2	1	2	2	2	2	2	2	2	2	2	2	2	2	1	2	2	2	2	2	2	2	2	1	2	2	2	2	2
9	1	0	1	1	1	1	1	1	1	1	1	2	2	2	2	2	2	2	2	2	2	2	2	2	2	2	2	2	2
10	2	2	2	2	2	2	2	2	2	1	2	2	2	2	2	2	2	2	2	2	2	2	2	2	2	2	2	2	2
11	1	0	1	1	1	1	2	0	2	1	1	1	1	0	2	1	1	1	2	0	1	1	0	2	1	1	1	2	0
12	0	0	1	1	0	0	0	0	0	0	0	1	0	0	0	0	0	1	0	1	1	0	0	0	0	0	1	0	1
13	2	2	2	2	2	2	2	2	2	2	2	2	2	2	2	2	2	2	2	2	2	2	2	2	2	2	2	2	2
14	2	2	2	2	2	2	2	2	2	2	2	2	2	2	2	2	1	2	2	2	2	2	2	2	2	1	2	2	2
15	2	2	2	2	2	2	2	2	2	2	2	2	2	2	2	2	2	2	2	2	2	2	2	2	2	2	2	2	2
16	2	2	2	2	2	2	2	2	2	2	2	2	2	2	2	2	2	2	2	2	2	2	2	2	2	2	2	2	2
17	2	2	2	2	2	2	2	2	2	2	2	2	2	2	2	2	2	2	2	2	2	2	2	2	2	2	2	2	2
18	2	2	2	2	2	2	2	2	2	2	2	2	2	2	2	3	2	2	2	2	2	2	2	2	3	2	2	2	2
19	2	2	2	2	2	2	2	2	2	2	2	2	2	2	2	2	2	2	2	2	2	2	2	2	2	2	2	2	2
20	2	2	2	2	2	2	2	2	2	1	2	2	2	2	2	2	2	2	2	2	2	2	2	2	2	2	2	2	2
X	1	1	1	1	1	1	1	1	1	1	2	2	1	1	1	2	2	1	2	1	2	1	1	1	2	2	1	2	1
Y	0	0	0	0	0	0	0	0	0	0	0	0	0	0	0	0	0	0	0	0	0	0	0	0	0	0	0	0	0
i(5q)	1	1	1	1	0	1	1	1	1	1	1	1	1	1	1	1	1	1	1	1	1	1	1	1	1	1	1	1	1
der(5;12)	2	2	2	2	2	2	2	2	2	2	2	2	2	2	0	1	2	2	2	2	2	2	2	0	1	2	2	2	2
der(11;12)	1	1	1	0	0	0	0	1	0	0	1	1	1	2	1	1	1	1	0	1	1	1	2	1	1	1	1	0	1
der(12;3;X)	1	1	1	1	1	1	0	1	0	1	0	0	1	1	0	1	0	0	0	0	0	1	1	0	1	0	0	0	0
der(X;3)	1	1	1	1	1	1	1	1	0	1	0	0	1	1	0	0	0	1	0	0	0	1	1	0	0	0	1	0	0
der(9;12?)	1	0	0	0	0	0	0	0	0	0	0	0	0	0	0	0	0	0	0	0	0	0	0	0	0	0	0	0	0
der(9)	0	0	0	0	0	0	0	0	0	0	0	0	0	1	0	0	0	0	0	0	0	0	1	0	0	0	0	0	0
der(2;5)	0	0	0	0	0	0	0	0	0	0	1	0	0	0	0	0	1	0	0	0	0	0	0	0	0	1	0	0	0
der(3;12)	0	0	0	0	0	0	0	0	0	0	1	0	0	0	1	0	0	0	1	1	0	0	0	1	0	0	0	1	1
dup(9)	0	1	0	0	0	0	0	1	0	0	0	0	0	0	0	0	0	0	0	0	0	0	0	0	0	0	0	0	0
der(6;12)	0	1	0	0	0	0	0	0	0	0	0	0	0	0	0	0	0	0	0	0	0	0	0	0	0	0	0	0	0
der(6)	0	1	0	0	0	0	0	0	0	0	0	0	0	0	0	0	0	0	0	0	0	0	0	0	0	0	0	0	0
der(3;11)	0	1	0	0	0	0	0	0	0	0	0	0	0	0	0	0	0	0	0	0	0	0	0	0	0	0	0	0	0
dup(8)	0	0	0	0	0	0	0	0	0	0	0	0	0	0	1	0	0	0	0	0	0	0	0	1	0	0	0	0	0
der(9;12;11)	0	0	0	1	0	0	0	0	0	0	0	0	0	0	0	0	0	0	0	0	0	0	0	0	0	0	0	0	0
der(i5(q);7)	0	0	0	0	1	0	0	0	0	0	0	0	0	0	0	0	0	0	0	0	0	0	0	0	0	0	0	0	0
der(7)	0	0	0	0	1	0	0	0	0	0	0	0	0	0	0	0	0	0	0	0	0	0	0	0	0	0	0	0	0
dic(9;11)	0	0	0	0	0	1	0	0	0	0	0	0	0	0	0	0	0	0	0	0	0	0	0	0	0	0	0	0	0
der(9;3?)	0	0	0	0	0	0	1	0	0	0	0	0	0	0	0	0	0	0	0	0	0	0	0	0	0	0	0	0	0
der(14)	0	0	0	0	0	0	0	0	0	0	0	0	0	0	0	0	1	0	0	0	0	0	0	0	0	1	0	0	0
der(X;3;9)	0	0	0	0	0	0	0	0	0	0	0	0	0	0	0	0	0	1	0	0	0	0	0	0	0	0	1	0	0
der(3;9)	0	0	0	0	0	0	0	0	1	0	0	0	0	0	0	0	0	0	0	0	0	0	0	0	0	0	0	0	0
der(X;9;12)	0	0	0	0	0	0	0	0	1	0	0	0	0	0	0	0	0	0	0	0	0	0	0	0	0	0	0	0	0
der(2)	0	0	0	0	0	0	0	0	1	0	0	0	0	0	0	0	0	0	0	0	0	0	0	0	0	0	0	0	0

**Table:** 

(e)							
C7 Karyotype	1	2	3	4	5	6	7
No. of Chrs.	39	41	41	40	42	41	42
Chromosomes	Chromosome Copy Number						
1	2	2	2	2	2	2	2
2	2	2	2	2	2	2	2
3	0	1	1	1	1	1	1
4	2	2	2	2	2	2	2
5	1	1	1	1	1	1	1
6	1	1	1	1	2	2	2
7	2	2	2	2	2	2	2
8	2	2	2	2	2	2	2
9	2	2	2	2	2	2	2
10	2	2	2	2	2	2	2
11	1	1	1	1	1	1	1
12	1	2	2	2	2	2	2
13	2	2	2	2	2	2	2
14	2	2	2	2	2	2	2
15	2	2	2	2	2	2	2
16	1	2	2	2	2	2	2
17	2	2	2	2	2	2	2
18	2	2	2	2	2	2	2
19	2	2	2	2	2	2	2
20	2	2	2	2	2	2	2
X	2	2	2	2	2	2	2
Y	0	0	0	0	0	0	0
dic(5;5;6)	1	0	0	0	1	1	0
der(3;11)	1	1	1	1	1	1	1
dic(5;6)	0	1	1	1	0	0	0
der(6;5)	0	1	1	0	0	0	1
der(3;12)	1	0	0	0	0	0	0
dic(6;16)	1	0	0	0	0	0	0
der(5;5)	0	0	0	0	1	0	0
der(5)	0	0	0	0	0	0	1

**Table:** 

(f)										
C8 Karyotype	1	2	3	4	5	6	7	8	9	10
No. of Chrs.	38	38	40	42	40	41	42	41	40	41
Chromosomes	Chromosome Copy Number									
1	2	2	2	3	2	2	2	2	3	2
2	2	2	2	2	2	2	2	2	2	2
3	1	0	1	1	1	0	1	1	1	1
4	2	2	2	2	2	2	2	2	2	2
5	1	1	1	1	1	1	1	1	1	1
6	2	2	2	2	2	2	1	2	2	2
7	2	2	2	2	2	2	2	2	2	2
8	2	2	2	2	2	2	2	2	2	2
9	2	2	2	2	2	2	2	2	2	2
10	2	2	2	2	2	2	2	2	2	2
11	1	1	1	0	1	1	1	2	1	1
12	1	0	0	1	0	2	0	0	0	0
13	2	2	2	2	2	2	2	2	1	2
14	2	2	2	2	2	2	2	2	2	2
15	2	2	2	2	2	2	2	2	2	2
16	2	2	2	2	2	2	2	2	2	2
17	2	2	2	2	2	2	2	2	2	2
18	2	2	2	2	2	2	2	2	2	2
19	2	2	1	2	2	2	2	2	2	2
20	2	2	2	2	2	2	2	2	2	2
X	2	2	2	2	2	1	2	2	2	2
Y	0	0	0	0	0	0	0	0	0	0
der(5,5)	0	1	1	1	1	0	1	1	1	1
der(3,12)	0	1	1	0	1	0	1	1	1	1
der(5,12)	0	0	1	1	0	2	1	1	0	1
der(11,12)	0	0	1	2	1	0	1	0	0	1
der(3,11)	0	0	0	0	0	1	0	0	0	0
der(6)	0	0	0	0	0	0	1	0	0	0
der(X)	0	0	0	0	0	0	1	0	0	0

**Table:** 

(g)									
C9 Karyotype	1	2	3	4	5	6	7	8	9
No. of Chrs.	40	40	39	41	40	41	41	40	41
Chromosomes	Chromosome Copy Number								
1	2	2	2	2	2	2	2	2	2
2	2	2	1	2	2	2	2	2	2
3	1	1	1	1	1	1	0	1	1
4	2	2	2	2	2	2	2	2	2
5	1	1	1	1	1	1	1	1	1
6	2	1	1	2	1	1	2	1	1
7	2	2	2	2	2	2	2	2	2
8	2	2	2	2	2	2	2	2	2
9	2	2	2	2	2	2	2	2	2
10	2	2	2	2	2	2	2	2	2
11	1	1	1	1	1	1	1	1	1
12	2	2	1	2	2	2	2	2	2
13	1	2	2	2	2	2	2	2	2
14	2	2	2	2	1	2	2	2	2
15	2	2	2	2	2	2	2	2	2
16	2	2	2	2	2	2	2	2	2
17	2	2	2	2	2	2	2	2	2
18	2	2	2	2	2	2	2	2	2
19	2	2	2	2	2	2	2	2	2
20	2	2	2	2	2	2	2	2	2
X	2	2	2	2	2	2	2	2	2
Y	0	0	0	0	0	0	0	0	0
der(3;11)	1	1	1	1	1	1	1	1	1
der(5;5)	0	0	0	0	1	1	1	0	1
dic(5;5;6)	0	1	1	1	0	0	0	1	0
dic(5;5;13)	1	0	0	0	0	0	0	0	0
dic(2;6)	0	0	1	0	0	0	0	0	0
dic(6;13)	0	0	0	0	1	0	0	0	0
dic(6;6)	0	0	0	0	0	1	0	0	0
der(3;6)	0	0	0	0	0	0	1	0	0
der(6)	0	0	0	0	0	0	0	0	1

**Table 4:** Clonalities of intact and of marker chromosomes of the neoplastic F10 rat clone and of six subclones C1, C2, C6, C7, C8 and C9.

Cytogenetic Analyses of a Neoplastic Primary Rat Clone F10 and Six Sub-Clones							
	F10 Stem Line	C1	C2	C6	C7	C8	C9
Avg Chr. No. ± SD	45.1 ± 11.8	42.7 ± 1.72	40.9 ± 1.52	41.6 ± 1.33	40.9 ± 0.99	40.3 ± 1.35	40.3 ± 0.67
Chromosomes	copy no (% clonal)						
1	2 (83)	2 (88)	2 (78)	2 (97)	2 (100)	2 (80)	2 (100)
2	2 (90)	2 (88)	2 (56)	2 (97)	2 (100)	2 (100)	2 (89)
3	1 (70)	1 (94)	1 (89)	1 (76)	1 (86)	1 (80)	1 (89)
4	2 (87)	2 (88)	2 (89)	2 (90)	2 (100)	2 (100)	2 (100)
5	1 (53)	1 (75)	1 (89)	1 (100)	1 (100)	1 (100)	1 (100)
6	2 (77)	2 (100)	2 (78)	2 (100)	1 (57)	2 (90)	1 (67)
7	2 (83)	2 (94)	1 (67)	2 (93)	2 (100)	2 (100)	2 (100)
8	2 (90)	2 (100)	2 (78)	2 (90)	2 (100)	2 (100)	2 (100)
9	2 (90)	1 (56)	1 (67)	2 (62)	2 (100)	2 (100)	2 (100)
10	2 (87)	2 (94)	2 (89)	2 (97)	2 (100)	2 (100)	2 (100)
11	1 (77)	1 (50)	1 (100)	1 (59)	1 (100)	1 (80)	1 (100)
12	1 (60)	0 (94)	0 (100)	0 (72)	2 (86)	0 (70)	2 (89)
13	2 (87)	2 (94)	2 (89)	2 (100)	2 (100)	2 (90)	2 (89)
14	2 (87)	2 (100)	2 (89)	2 (93)	2 (100)	2 (100)	2 (89)
15	2 (90)	2 (100)	2 (78)	2 (100)	2 (100)	2 (100)	2 (100)
16	2 (90)	2 (100)	2 (100)	2 (100)	2 (86)	2 (100)	2 (100)
17	2 (87)	2 (94)	2 (100)	2 (100)	2 (100)	2 (100)	2 (100)
18	2 (83)	2 (100)	2 (100)	2 (93)	2 (100)	2 (100)	2 (100)
19	2 (87)	2 (100)	2 (100)	2 (100)	2 (100)	2 (90)	2 (100)
20	2 (90)	2 (94)	2 (100)	2 (97)	2 (100)	2 (100)	2 (100)
X	2 (90)	1 (56)	2 (100)	1 (69)	2 (100)	2 (90)	2 (100)
Y	0	0	0	0	0	0	0
der(3;12)	1 (40)	1 (44)	1 (100)	1 (23)	1 (14)	1 (70)	-
i(5q)	1 (33)	1 (81)	1 (100)	1 (97)	-	-	-
der(5;11)	1 (30)	-	-	-	-	-	-
der(3;11)	1 (20)	-	-	1 (3)	1 (100)	1 (10)	1 (100)
der(5;12)	1 (13)	2 (75)	-	2 (86)	-	1 (50)	-
der(5;5)	1 (20)	-	1 (11)	-	1 (14)	1 (80)	1 (44)
der(5;9)	1 (3)	-	1 (89)	-	-	-	-
der(11;12)	1 (7)	1 (50)	1 (100)	1 (66)	-	0 (50)	-
der(5;7)	1 (3)	1 (6)	1 (56)	-	-	-	-
der(5)	1 (7)	-	1 (11)	-	1 (14)	-	-
der(X;3)	-	1 (44)	-	1 (52)	-	-	-
